# Noncoding RNA and Alcohol Use Disorder: A Scoping Review of Current Research and Knowledge Gaps

**DOI:** 10.35946/arcr.v45.1.06

**Published:** 2025-06-20

**Authors:** Deepa Upreti, Rosaline P. Kumar, Justin B.J. Chen, Sneha L. Sonti, Abigail V. Bowring, Sheila W. Green, Rajesh C. Miranda

**Affiliations:** 1 Department of Neuroscience and Experimental Therapeutics, Texas A&M School of Medicine, Bryan, Texas; 2 Department, Medical Sciences Library, Texas A&M University Libraries, College Station, Texas

**Keywords:** alcohol, RNA, microRNAs, long noncoding, circular RNA, alcohol-related disorders

## Abstract

**BACKGROUND:**

Alcohol use and misuse can result in substantial disease burden and mortality, with significant public health and social costs. The need for better diagnoses and medications development for all conditions associated with alcohol use emphasizes the need for research into underlying molecular mechanisms. Noncoding ribonucleic acids (ncRNAs) are an explanatory mechanism for transducing environmental effects into cells and tissues. ncRNAs are regulatory RNAs that are diverse in size and function and greatly outnumber protein-coding RNAs in mammals. ncRNAs may play a major role in the pathogenesis and consequences of alcohol use and misuse, and studies in this area could pave the way to developing novel methods of diagnosis and therapy.

**OBJECTIVES:**

This scoping review examines the extent, range, and nature of the research linking ncRNAs to alcohol, with a focus on identifying gaps in the existing literature.

**ELIGIBILITY CRITERIA:**

This scoping review followed the “Preferred Reporting Items for Systematic Reviews and Meta-Analyses extension for Scoping Reviews.” Peer-reviewed journal articles for all species, including human, animal, or cells, published until December 2023, were included.

**SOURCES OF EVIDENCE:**

Publications were retrieved using keyword searches in three online databases: Medline (Ovid), Embase (Ovid), and Academic Search Ultimate (EBSCO).

**CHART METHODS:**

Identified articles were imported in Covidence systematic review software for screening. Each article was evaluated by at least two independent reviewers, and only those receiving votes from both were included in the review. Key findings were then extracted from the included studies, further analyzed, and summarized in a table and figures using Microsoft Excel. Details, including year of publication, species, sex, sample type, and sample processing methods for different types of ncRNAs (i.e., microRNAs [miRNAs], long noncoding RNAs [lncRNAs], circular RNAs [circRNAs]) were also reported.

**RESULTS:**

In total, 3,358 studies were identified and imported in Covidence. After removal of duplicates, 1,937 studies were processed for title and abstract screening, and 400 studies were subsequently selected for full-text screening. From these, 338 studies were included in the scoping review. In total, 3,020 initially captured studies were excluded. Among all ncRNAs, miRNAs were the most frequently investigated, followed by lncRNAs and circRNAs. Whereas many studies investigated ncRNA associations with alcohol phenotypes, mechanistic studies were more limited. Studies spanned pathologies related to alcohol use across tissues and organs, including liver, brain, heart, pancreas, placenta, gastrointestinal system, muscle, and bone. However, key variables, including biological sex, age, and genetic variation, were not adequately addressed. The analyses uncovered significant gaps in the research literature, relating primarily to underlying mechanisms.

**CONCLUSIONS:**

The field of ncRNA research in pathologies associated with alcohol use is still emerging. Given the enormous sizes and species variations of mammalian ncRNA genomes, a significant amount of research is needed to identify relevant ncRNAs in different organs, and at all stages of pathology, and to identify underlying mechanisms. Initial studies show promise that ncRNA research could significantly improve the diagnosis and treatment of alcohol use disorder.

According to the 2022 National Survey on Drug Use and Health, 221.3 million people ages 12 and older in the United States (78% in this age group), reported that they drank alcohol at some point in their lifetime.^1^ In this group, 28.8 million adults ages 18 and older (11% in this age group) had alcohol use disorder (AUD) in the past year. AUD has been defined as a chronic relapsing disorder characterized by a compulsion to consume alcohol, and the onset of negative mood states if alcohol is unavailable.^2^ AUD contributes to a range of adverse mental and physical health problems, including liver and other organ damage, chronic inflammation, increased susceptibility to infections, and cancers. The effects of alcohol can also be transmitted from one generation to the next, because alcohol exposure in the developing fetus can result in fetal alcohol spectrum disorders (FASD). Additionally, AUD is linked to increased mortality rates,^3^ and even moderate levels of alcohol consumption that do not meet the diagnostic criteria of AUD are also associated with increased disease burden.^4^ To date, only three pharmacological agents—disulfiram, naltrexone, and acamprosate—have been approved for the treatment of AUD itself,^2^ although treatment also includes management of end-organ damage and other consequences of alcohol use and misuse. However, according to recent data, few people receive medication-assisted treatment for AUD. For example, in 2023, only 2% of people ages 12 and older with past-year AUD had received medication-assisted treatment in the past year.^5^ The significant public health burden of AUD highlights the urgent need to identify mechanisms that contribute to the emergence of AUD and their secondary outcomes, and to find novel interventions to manage all aspects of AUD.

At the molecular level, most studies on alcohol use and AUD, and their secondary pathologies, have focused on the contributory role of genes that encode proteins. However, evidence accumulated over the last 30 years suggests that this emphasis on protein-coding genes, which comprise about 2% of the mammalian genome, may limit our understanding of alcohol-associated pathology. Moreover, the number of protein-coding genes has not changed with mammalian evolution. For instance, GENCODE™,^6^ an extensive gene annotation initiative that systematically identifies and characterizes both protein-coding and noncoding DNA sequences within the human genome, annotates 19,411 protein-coding genes in the human genome (release version 46), although the total number of annotated DNA sequences that encode ribonucleic acid (RNA) transcripts currently stands at 63,086. In fact, the vast majority of the mammalian genome encodes RNA transcripts that are not translated into proteins.^7^ Therefore, human complexity, including complex phenotypic traits associated with AUD, cannot be explained solely by protein-coding genes. Increasing evidence shows that these noncoding RNA (ncRNA) molecules, which do not encode proteins, can nevertheless regulate gene expression at the transcription and translation levels. They contribute to layers of epigenetic regulation (i.e., regulation of gene activity without changing the DNA sequence) that transduce environmental influences into cells and tissues, resulting in phenotypic variation.^8^ Many studies have now explored such regulatory roles for ncRNAs.^9^ It is clear from studies in other disease pathologies that ncRNAs have the potential to help with early diagnosis of AUD and consequent pathologies and novel RNA-type drugs to treat AUD. Therefore, a review of the available research on AUD-related ncRNAs to determine the current state of knowledge may uncover unexpected gaps in the knowledge base and point to avenues for further exploration.

A comprehensive review of the different types of ncRNAs and their functions is beyond the scope of the current review, but may be found elsewhere (e.g., Mattick et al. 2023^10^,^11^ and Nemeth et al. 2024^11^). Briefly, ncRNAs are a diverse group of RNA molecules that have been somewhat arbitrarily divided into two categories—short ncRNAs and long ncRNAs.^7^ Each category contains several subtypes that have unique characteristics and functions, making them important in understanding how alcohol affects the body and brain.

Short ncRNAs are typically less than 200 nucleotides in length and together play critical roles in gene expression, gene silencing, and cellular regulation. They include the following major subtypes:

- microRNAs (miRNAs) are about 17–25 nucleotides in length; they bind to regulatory regions (i.e., the 3′-untranslated regions [UTRs]) of target messenger RNAs (mRNAs) to destabilize these mRNAs and repress protein translation.
- Transfer RNAs (tRNAs) deliver specific amino acids to the ribosome during protein synthesis, ensuring accurate translation of mRNA.
- Small nuclear RNAs (snRNAs) are essential components of the spliceosome, facilitating the precise removal of noncoding introns from pre-mRNA.
- Short ribosomal RNAs (rRNAs), such as 5S rRNAs that, together with their long rRNA partners, form the structural and catalytic core of ribosomes, driving protein synthesis by decoding mRNA.
- Small nucleolar RNAs (snoRNAs) guide the chemical modification of other RNAs, such as rRNAs and snRNAs, to enhance their stability and functionality.
- Small interfering RNAs (siRNAs) mediate RNA interference by degrading complementary mRNA sequences, thereby silencing specific genes.
- Piwi-interacting RNAs (piRNAs) protect genomic integrity in germ cells by silencing transposable elements and regulating epigenetic modifications.

Long ncRNAs (lncRNAs), in contrast, are more than 200 nucleotides in length and can range up to several thousand nucleotides. They include subtypes such as antisense RNAs, enhancer RNAs (eRNAs), long intergenic noncoding RNAs (lincRNAs), and long rRNAs (18S and 28S) among others. Unlike short ncRNAs, lncRNAs can regulate gene expression at multiple levels, including chromatin remodeling, transcription, and post-transcriptional processing. For instance, some lncRNAs act as molecular scaffolds, bringing together proteins and DNA to influence gene activity, while others act as decoys to sequester transcription factors or RNA-binding proteins.

In addition to short and long ncRNAs, circular RNAs (circRNAs) represent a distinct class of ncRNAs characterized by their covalently closed loop structures, which lack 5′ and 3′ ends. circRNAs often act as molecular “sponges” for miRNAs, sequestering them to prevent their interaction with target mRNAs, and they can also interact with proteins to modulate cellular pathways. Their stability and unique regulatory roles make circRNAs an emerging focus in understanding gene regulation and disease mechanisms. Together, short, long, and circular ncRNAs represent a versatile toolkit for regulating genetic information, with their diverse functions making them central to both normal physiology and disease mechanisms.

The first ncRNA, a tRNA, was discovered in 1965,12 but it was not until the 1990s that research on ncRNAs started to advance with the discovery of lncRNAs XIST13 and H1914 and the discovery of the first miRNA, lin4, in nematodes.15–17 Initially, more studies were focused on constitutive small ncRNAs, such as tRNAs, snoRNAs, and snRNAs, and large rRNAs.18 However, miRNAs have become a major research focus across a wide range of biological processes, including cancers and infectious diseases, with more than 173,000 citations in PubMed from 1993 to 2024. Unsurprisingly, as documented below, a majority of research on ncRNAs in the pathogenesis of AUD similarly focuses on miRNA mechanisms, with relatively poor representation of other ncRNAs in the research literature.

This scoping review of the existing literature aimed to enhance understanding of the research supporting a role for ncRNAs in the pathogenesis of ethanol across tissues and developmental stages. Consistent with the focus of a scoping study,^19^,^20^ the primary aim was to identify gaps in knowledge.

The following key questions were addressed:

1. What is the scope of the literature on the role of ncRNAs in AUD?
2. To what extent does the literature cover AUD pathology across tissues and organs, given that alcohol has systemic effects throughout the body, as well as effects across the lifespan? To capture data across the lifespan, this review included studies on both prenatal and adult alcohol exposure and their outcomes.
3. How do ncRNAs contribute to the clinical consequences of alcohol use and misuse?
4. What evidence supports the use of ncRNAs as biomarkers in the diagnosis of AUD and related pathological outcomes, including cancer, liver disease, and fetal alcohol effects? Are such studies useful for identifying therapeutic targets and monitoring treatment efficacy?
5. Which specific gaps in knowledge of ncRNA biology in AUD merit further research?

## Methods

### Protocol Registration

The protocol for this study was based on the 22-item Preferred Reporting Items for Systematic Reviews and Meta-Analyses extension for Scoping Reviews (PRISMA-ScR).^21^,^22^ The protocol was not pre-registered but can be obtained upon request from the corresponding authors.

### Eligibility Criteria

Peer-reviewed journal articles were included if they were: (a) primary research with humans, animals, or cells; (b) explorations of the role of ncRNA (miRNA, lncRNAs, circRNA, or other ncRNAs) as intermediaries for developmental and other processes in the context of alcohol use; (c) published up to December 2023; and (d) written in English. Papers were excluded if they were reviews, editorials, or other non-primary research, or non-English-language publications. The underlying rationale for the above criteria is that alcohol is a pleiotropic drug that alters brain circuits but acts also as a teratogen and results in multi-organ disease, including organ damage and cancer. The search strategy was intended to capture research on ncRNAs across alcohol’s diverse systemic effects.

### Search Strategy

To identify potentially relevant papers, the authors searched the following bibliographic databases for articles published prior to December 2023: Medline (Ovid), Embase (Ovid), and Academic Search Ultimate (EBSCO). An experienced librarian (S.G.) developed the search strategies, which were further refined through team discussion. Search concepts for miRNAs, circRNAs, ncRNAs, alcohol/ethanol, and human or animal populations were searched through keywords and thesaurus headings as appropriate for the database. Search strategies were developed and refined through several iterations. Initially, the list of titles was prepared from the randomly selected papers in PubMed^®^. Three reviewers assigned to this project phase then met to discuss the results and refine search strategies. The final search strategies used are outlined in Tables 1 and 2. The final search results were entered into a Covidence database for de-duplication and facilitation of the PRISMA methodology.

### Screening and Selection Criteria

A total of 3,358 studies were imported in Covidence systematic review software (Veritas Health Innovation, Melbourne, Australia. Available at www.covidence.org). After removing 1,392 duplicates identified by the software and an additional 29 manually identified duplicates, 1,937 studies remained for title and abstract screening. During this screening, any additional duplicates, review articles, or studies with only abstracts were manually removed. Studies that only addressed ncRNAs without direct association with, or manipulation of, alcohol exposure were excluded. All experimental species (e.g., mouse, rhesus monkey, human) and disease conditions (e.g., hepatitis, inflammation, cancer) were included. Two reviewers independently screened each title and abstract in Covidence. Any disagreements between two reviewers were resolved by a third reviewer, whose decision was final. After the title and abstract screening phase, 400 studies remained and were moved to the full-text review stage, which used a similar approach as the title/abstract screening. During the full-text review, studies that did not meet the inclusion criteria established during the title and abstract screening phase, as mentioned above, were excluded. Further, the selection focused on organ systems that are the best documented targets of alcohol consumption, including liver, cardiovascular system, brain, and fetal development; studies of tissues of the reproductive system were excluded due to human resource and time constraints. Also excluded were citations with only an abstract but no full text available, studies that had no association between ncRNAs and alcohol in any disease condition, those that only screened for ncRNAs in any disease condition without a direct link to alcohol, studies that had no PubMed IDs, and any studies that were solely simulations or modelling based on secondary analysis of existing data curated in databases. Although all types of ncRNAs—including miRNAs, circRNAs, lncRNAs, eRNAs, tRNAs, and rRNAs—were included in the literature search, the subsequent discussion will focus on miRNAs, lncRNAs, and circRNAs because either no studies, or only a single study, addressed the other types of ncRNA or because, as in the case of eRNAs, publications were not uniformly captured due to the unsettled nature of the ncRNA terminology in the literature. This comprehensive approach resulted in the inclusion of 338 studies for final analysis. A list of the references for all the included studies is available upon request from the corresponding authors.

**Figure 1:**
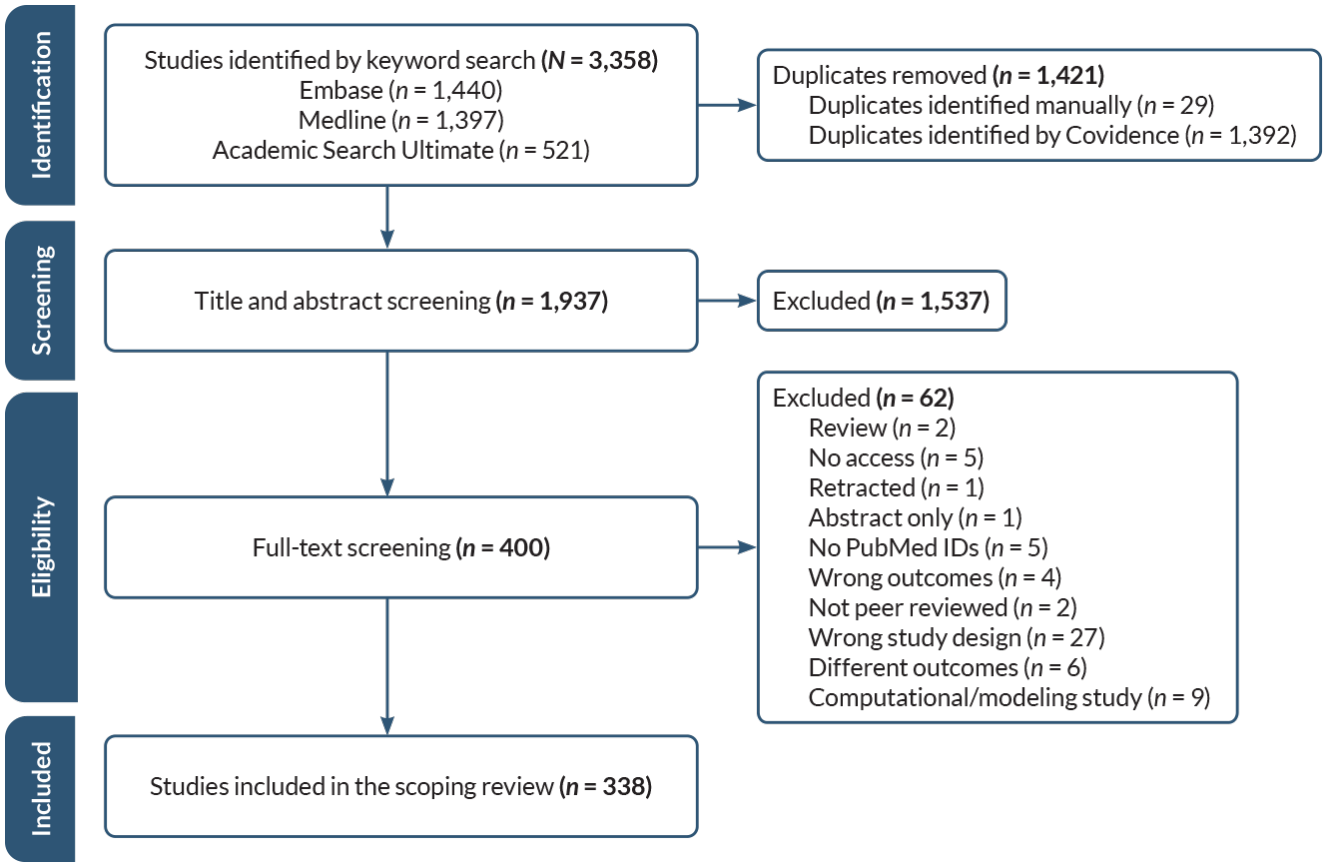
The PRISMA flow diagram of the study selection process for alcohol use and noncoding RNAs.

The PRISMA flow diagram of the scoping review (see Figure 1) was created using Covidence following the PRISMA guidelines.^21^,^22^ The objective for this scoping review was to address the current research and identify knowledge gaps in ncRNA in association with AUD.

### Data Extraction, Characterization, and Analyses

Details extracted from each full text screen included the author and year of the publication as well as the species (humans, mice, rats, zebrafish, or cell), sex (male, female, or both), and sample type (e.g., brain, liver, blood, saliva, embryo tissue, or cell culture) that were studied. Also charted were sample processing methods, such as RNA extraction method (e.g., Trizol, miRNeasy kit, mirVana miRNA isolation kit, RNeasy kit, guanidinium thiocyanate/phenol/chloroform method); method of ncRNA analysis (e.g., RNA sequencing, quantitative reverse transcription polymerase chain reaction [qRT-PCR], reverse transcription PCR [RT-PCR], miRNA 4.0 Arrays, miRCURY LNA Microarray kit); and biomarker analysis, including reported “area under the curve” (AUC) value from “Receiver Operator Characteristic” (ROC) analysis, when available.

All characteristics were extracted using Microsoft Excel 2024. Studies were grouped by the types of ncRNAs (i.e., lncRNA, circRNA, miRNA, eRNA, rRNA). Appendices 1 to 5 summarize the characteristics listed above along with the key findings of each study. For lncRNAs and circRNAs, key findings from all relevant studies are presented in one table. However, because of the extensive literature, studies were categorized by specific disease conditions for miRNAs. The number of the studies in the extracted Excel data sheet and in Covidence were counted and matched, and studies that were missed while extracting the data were noted. Any identified duplicates and articles without PubMed IDs were manually removed from the study.

The NIH iCite bibliometrics analytic platform was used to assess the impact and relevance of the included studies.^23^ Together with the data tables, this provided a comprehensive overview of the current state of research on ncRNAs in AUD and related pathologies, uncovered gaps in knowledge, and suggested directions for future studies. Sunburst figures that visually represent gaps in knowledge for the different types of ncRNA are also available from the authors.

## Results

### Selection of Sources of Evidence

The systematic search of the Embase, Medline, and Academic Search Ultimate databases yielded a total of 3,358 articles (see Figure 1). Specifically, the Embase search resulted in 1,440 articles, the Medline search resulted in 1,397 articles, and the Academic Search Ultimate search resulted in 521 articles. After removing duplicates, 1,937 unique articles remained for screening. Initial screening based on title and abstract exclusion criteria resulted in the exclusion of 1,537 studies. Subsequently, 400 full-text articles were assessed for eligibility. Following a detailed assessment against predefined inclusion and exclusion criteria, 338 studies were included in the study for qualitative analysis, and 3,020 initially captured studies were excluded.

### Study Characteristics

The 338 studies selected for qualitative analysis included a broad range of topics relevant to the research question. These studies were published between 2007 and December 2023 (the end of the assessment period) and included experimental as well as observational studies across a range of AUD-relevant disease states. The data in these studies were obtained with multiple species, sample types, and extraction and assessment methodologies. The characteristics of all included articles are summarized in Table 3.

Based on the studies collected in this review, research on the role of ncRNA in the pathophysiology of alcohol use and misuse began to emerge in 2007. However, significant and vigorous investigations into this area only appeared in the literature around 2012, with more than 10 studies reported in that year alone. Based on the iCite analysis, studies in human populations are well represented in the literature, along with studies in animal models. The number of publications reported per year has been steadily growing, reflecting increased interest in this field of ncRNAs and AUD research. The impact of these publications has also been high. Approximately 21% of the publications (72 articles) had a relative citation ratio (RCR, a field- and time-normalized citation rate, benchmarked to a score of 1.0 for the typical NIH-funded paper in that field^23^) of more than 2.0, with a maximum score of 14.9. This indicates that these publications were cited between two and more than 14 times more than other NIH-funded publications in the field. The median RCR for these 72 articles was 3.23. According to the iCite analysis, the selected papers collectively had an “approximate potential to translate” (APT) score of about 29%, and about 14% of the papers were cited at least once in a clinical paper, mainly in the field of alcohol-related liver disease or neurobiological basis of AUD, suggesting an emerging “bench-to-bedside” translational outcome.

Among the ncRNAs, miRNAs were most studied (86%) followed by lncRNAs (11%) and circRNAs (3.0%). Other types of ncRNAs each constitute a minimal percentage of studies and are therefore not discussed in this review. Regarding the sex distribution among the study subjects, males (42%) were more frequently studied than females (8%). A considerable proportion (31%) of studies involved both sexes, whereas a notable percentage (19%) of studies did not report sex-specific data. This highlights a gap in reporting standards across studies and emphasizes the importance of considering sex as a critical variable. In terms of model organisms and study subjects, mice (34%) were studied more often than rats (22%), humans (30%), or cells (7%). A few studies (about 6%) were conducted in other organisms, including invertebrates (drosophila), teleosts (zebrafish), other mammals (sheep), and non-human primates (macaques). Model diversity is important, since similarity of outcomes in diverse model organisms increases the translational relevance of these studies. Moreover, 36% of the studies used some combination of mice, rats, cells, and human tissues, which further emphasizes the cross-species translational potential of these studies. Finally, a wide range of samples were used for the analysis of ncRNAs, including blood/plasma/serum (14%), brain (27%), and liver/liver cells (20%). Other sample types included cells, embryos, and various tissues such as heart, lung, and intestinal epithelial cells.

Methods of RNA extraction in the individual studies were also charted. The Trizol reagent was the most commonly used method (32%), followed by the miRNeasy kit (17%), and mirVana isolation kit (13%). The traditional Trizol (guanidium isothiocyanate-phenol) reagent method is historically well-validated and facilitates the separation of RNA, DNA, and protein from a single sample by phase separation methodologies; however, it may result in loss of small RNAs. In contrast, other methodologies that couple an organic extraction step common to Trizol with RNA recovery on silica/glass fiber substrates (miRNeasy and mirVana) result in better retention of small RNAs. Several other extraction methods were used less frequently, and 9% of the studies did not report their RNA extraction method. Overall, a diverse range of approaches were used in ncRNA analysis. A majority of studies used qRT-PCR assays (51%) and related variants; RT-PCR (12%); or a combination of techniques (13%) that reflects the usage of an integrative approach for more robust analyses. The remaining studies used a variety of analytic methods. However, more than 1% of the studies lacked documentation of techniques used to analyze ncRNA. This is concerning, because methodological details are essential for documenting the integrity and trustworthiness of research outcomes.

## Analysis of ncRNA Classes

In the context of AUD, ncRNAs have gained attention for their ability to influence key pathways involved in brain function, alcohol metabolism, and immune responses. In the last 15 years, a substantial body of research has accumulated, demonstrating that ncRNAs are indeed sensitive to ethanol, and ethanol treatment induces changes on their transcript levels *in vivo* and *in vitro*, contributing to phenotypical variations that underlie the emergence of AUDs and their associated pathologies. Most of this research has focused on miRNAs, lncRNAs, and circRNAs; very little to no research has been conducted on other small ncRNAs, which will therefore not be discussed here.

### miRNAs

The first miRNA, lineage-4 (lin-4), was discovered in the 1990s as a critical regulator of nematode development.^15^–^17^ The discovery that all cells and tissues had the capacity for RNA interference (RNAi)—that is, inhibition of translation with small RNAs^24^—and the fact that the miRNA Let-7/Lethal-7^25^,^26^ was ubiquitously expressed in all eukaryotes were key drivers for research into miRNA biology. The next stage of discovery of miRNA research was the almost simultaneous discovery of machinery for miRNA processing. This includes the nuclear RNAse Drosha^27^,^28^ along with the double-stranded RNA-binding protein Di-George Syndrome Critical Region-8 (DGCR8),^27^,^29^ which processes kilobase-length primary miRNAs (pri-miRNA) into an about 70 nucleotide hairpin-structured pre-miRNA; Exportin-V, which transfers the pre-miRNA to the cytoplasm;^30^ DICER, which processes the pre-miRNAs in the cytoplasm into mature miRNAs; 31,32 and the argonaute (Ago) family^33^,^34^ that act as the key chaperones of the RNA-induced silencing complex (RISC). Following DICER cleavage, which yields a short double-strand RNA molecule, one strand, termed the “sense” or “guide” strand (usually, but not always, the 5′ strand) is loaded onto Ago proteins in the RISC complex, while the antisense strand, or “passenger miRNA” (usually, but not always, the 3′ strand) is generally assumed to be degraded. Finally, rules for the predictable and orderly behavior of miRNAs in silencing gene expression—including the importance of 5′ seed sequence homology (six to eight nucleotides at the 5′ end of a miRNA that exhibit reverse complementarity to a target region within the 3′UTRs of mRNAs), a mid-region of non-homology, and 3′ sequence pairing—are important determinants of mRNA stability, translation, and other processes.^35^–^38^ miRNAs also can be post-transcriptionally processed from lncRNAs. An example is miR-9, an evolutionarily ancient miRNA that was expressed in the first bilaterally symmetrical organisms and is important for brain development; in mammals, it is processed from one of three separate and unique lncRNAs (pri-miR-9-1, pri-miR-9-2, and pri-miR-9-3).^39^ In contrast, other miRNAs, such as miR-335, are encoded in the intron of protein-coding genes (sometimes termed miRtrons^40^) and have evolved more recently.

The first study implicating miRNAs in ethanol pathology was published in 2007.^41^ This scoping review identified an additional 289 papers published since then, comprising almost every aspect of AUD pathology, from brain circuits, organ toxicity, and cancer to pregnancy effects and developmental outcomes. This field of ncRNA biology has been the most thoroughly explored, comprising about 86% of all publications. Nevertheless, even here, significant gaps in our knowledge remain. The following sections summarize information and knowledge gaps regarding the role of miRNA in various conditions related to AUD.

#### Cancer

Due to their accessibility for assessment in body fluids and high biological stability, miRNAs have gained significant attention as important regulatory molecules and as biomarkers for tumor development, progression, and response to treatment in cancer biology.^42^ A review of studies investigating the role of miRNAs in different types of cancer associated with alcohol use showed that most of the studies were conducted in human samples, although a few were conducted in mice or cell cultures (see Appendix 1). Key findings are that specific miRNAs are indeed differentially up- or down-regulated in response to alcohol exposure in a variety of cancers, such as hepatocellular carcinoma, head and neck squamous cell carcinoma, and esophageal squamous cell carcinoma. Differential miRNA expression in cancers associated with alcohol use holds a promise that miRNAs can be used as biomarkers for early detection and diagnosis of alcohol-related cancers. However, vigorous further validation of these miRNAs as potential biomarkers is needed. Furthermore, miRNAs play a key regulatory role at both transcriptional and post-translational levels (see below), and these roles are not yet fully understood in cancer biology. Moreover, the mechanisms that control miRNA expression in cancer warrant further investigation. For instance, in oral squamous cell carcinomas, shorter survival was associated with miR-34b/c through promoter methylation,^43^ highlighting the importance of studying epigenetic modifications and gene expressions in cancer associated with alcohol.

#### Musculoskeletal health

Several studies demonstrated that expression levels of specific miRNAs had an impact on adults with musculoskeletal conditions such as osteopenia, osteonecrosis, muscle atrophy and dysfunction, as well as fractures and healing (see Appendix 2A). These studies reported that alcohol exposure led to a decrease in miRNA levels such as miR-4286 and miR-136-3p, which in turn led to impaired osteogenic differentiation.^44^,^45^ In contrast, miR-31 was highly expressed in ethanol-induced osteonecrosis tissues, and decrease of miR-31 induced by a tumor necrosis factor alpha (TNF-alpha) inhibitor could activate the SATB2/RUNX2 regulatory pathway, leading to increased osteogenic differentiation.^46^ These expression patterns in bone disorders open an avenue to investigate miRNAs further as therapeutics for alcohol-induced musculoskeletal disorders. Agomirs—synthetic molecules that mimic miRNA—are a potential means for therapeutic interventions in alcohol-induced bone disorders. An agomir for miR-19a-3p was able to improve alcohol-impaired fracture healing, while a miR-136-3p agomir was able to ameliorate downregulation of miR-136-3p in alcohol-induced osteopenia, which suppressed osteogenic differentiation.^45^,^47^

Furthermore, miRNAs such as miR-1, miR-127-3p, miR-483-5p, miR-483-3p, miR-628-3p, and miR-885-5p have been explored as diagnostic biomarkers for alcohol-induced osteonecrosis of the femoral head^48^ and have shown promise for early detection and diagnosis of alcohol-induced musculoskeletal disorders. A key future goal will be to ascertain whether such signatures are unique to alcohol’s effects on bone or a shared signal with other organ responses to alcohol. Finally, in a zebrafish model, miR-140-3p and miR-146a were shown to associated with Notch signaling and muscle differentiation, advancing a role for specific signaling pathways as a mechanistic intermediary.^47^ These studies highlight the crucial role of miRNAs in musculoskeletal health, offering insights into potential therapeutic targets and diagnostic markers; however, further studies can provide clinical validation, mechanistic insight, and functional interactions.

#### Cardiovascular disease

The interplay between alcohol exposure, cardiovascular disease, and miRNAs also was assessed (see Appendix 2B). Several studies showed differential miRNA expression with alcohol exposure in cardiovascular tissues. Specifically, miR-155-5p has been shown to play a role in alcohol-associated cardiovascular pathophysiology. miR-155 is a multifunctional miRNA that mediates several pathophysiological processes in cardiovascular diseases such as coronary artery disease, heart failure, and diabetic heart disease.^49^ For example, studies found that miR-155-5p appeared to regulate ethanol-induced myocardial insulin resistance, affecting mammalian target of rapamycin (mTOR) pathways.^50^,^51^ Moreover, miR-155-5p increased in serum following alcohol consumption, and this increase correlated with cellular apoptosis in ischemic rats as well as rats with chronic alcohol exposure.^52^ Finally, miR-155-5p played a significant role in inflammation mechanisms induced by alcohol that potentially affect cardiovascular health.^53^ miRNAs have also been explored as biomarkers for cardiovascular diseases; however, this review did not find any studies reporting on miRNAs as biomarkers or therapeutics for alcohol-related cardiovascular disease and risks.

#### Gastrointestinal injury

Chronic alcohol ingestion alters the gut microbiome composition and increases intestinal permeability and inflammation—factors that could contribute to damage in other organs within the enteric portal circulation, such as the liver (for recent reviews, see Bajaj 2019^54^ and DiVincenzo et al. 2024^55^). miRNAs control a variety of cellular processes, including proliferation, cell death, and integration of enterocytes into a functional epithelial barrier.^56^ They also affect gut inflammation and the composition of the microbiome.^57^ A number of studies have explored the link between alcohol consumption, miRNAs, and intestinal barrier function (see Appendix 2C). Two studies by Tang et al.^58^,^59^ focused on miR-212 as a mechanism underlying reduced ZO-1 protein and enhanced alcohol-induced gut leakiness. An elevation of miR-9-5p was found in alcohol-induced gastric ulcer, which was alleviated by treatment with glycopeptides from the fungus Paecilomyces sinensis (CPS-II) that reduced inflammation and restored mucosal integrity.^60^ This shows that miRNAs may potentially be used as targets for therapeutic intervention to maintain intestinal homeostasis in alcohol-induced gut barrier dysfunction.

Other studies have highlighted the dysregulation of several miRNAs with alcohol exposure, such as miR-141, miR-21, miR-145, miR-155-5p, and miR-146a-5p. These miRNAs play roles in mediating inflammatory responses, apoptosis, oxidative stress, and barrier integrity in gastrointestinal and liver tissues.^61^–^64^ A key consideration when assessing the potential therapeutic utility of these miRNAs is whether target-organ specificity can be achieved, since at least some of these gut-active miRNAs (e.g., miR-21 and miR-9-5p) also have been implicated in alcohol effects in other tissues and pathologies.

#### Pancreatic and metabolic disease

Several studies have explored the relationship between alcohol, pancreatic and metabolic diseases, and miRNAs (see Appendix 2D). Alcohol use is a risk factor for pancreatitis in both men and women.^65^ A pilot study in human populations documented the differential expression of 150 miRNAs in serum obtained from patients with alcohol-associated chronic pancreatitis.^66^ The authors of that study used informatics approaches to link differentially expressed miRNAs to inflammatory processes, which they suggested were part of the pathology of the condition. In a mouse model, alcohol was shown to upregulate the secretion of both connective tissue growth factor 2 and miR-21 by pancreatic stellate cells; moreover, the miRNA and growth factor were part of a positive feedback loop that promoted collagen production and pancreatic fibrosis.^67^

In contrast with pancreatitis, the link between alcohol consumption and diabetes is complex: moderate levels of consumption have been associated with decreased risk for diabetes, whereas heavy binge-like consumption patterns (defined as five or more U.S. standard drinks per occasion^68^) are associated with increased risk for diabetes.^69^,^70^ One study profiled circulating miRNAs in patients with type 2 diabetes and reported that miR-330, which was elevated in diabetes, also was elevated in persons with AUD.^71^ Again, more research will help to better understand the mechanisms involved and to validate miRNAs as biomarkers for alcohol-associated pancreatitis and metabolic disease.

#### Liver disease

Among the studies included in this scoping review that investigated the role of miRNAs in AUD and associated pathologies across organs, the majority (38%) focused on alcohol-associated liver disease (ALD) in adult human or animal models. ALD is considered the significant contributor to chronic liver diseases, including alcohol-associated fatty liver disease/hepatic steatosis, which can progress to more severe conditions such as alcohol-associated steatohepatitis (ASH), alcohol-associated hepatitis (AH), fibrosis, and cirrhosis. As seen in other pathological conditions, several miRNAs were found to be differentially expressed in patients with ALD or animal models, including upregulation of miR-122, 72–74 miR-155, 73,75,76 miR-21,^77^,^78^ and miR-34a^79^,^80^ (see Appendix 2E). miR-122 is a liver-specific miRNA that is highly expressed in the liver and plays a crucial role in metabolism and cell growth and death.^81^ miR-122 along with miR-155 is suggested to be a potential biomarker for liver damage and inflammation in ALD.^73^ Furthermore, miR-21 and miR-34a are involved in regulating pathways related to cell proliferation, apoptosis, and survival, impacting the progression of ALD.^78^,^79^ Circulating miRNAs, such as miR-513-3p, miR-571, and miR-652, which are found in bodily fluids and serve as stable, non-invasive biomarkers for various physiological and pathological processes, were also differentially expressed in patients with chronic liver disease and liver cirrhosis, demonstrating high diagnostic accuracy for cirrhosis progression.^82^ Additionally, miR-203 showed a protective role in alcohol-associated fatty liver disease;^83^ miR26a protected against ethanol-induced hepatic steatosis and liver injury;^84^ and miR-205 reduced ALD progression.^85^ This suggests that these miRNAs could potentially be used as therapeutic targets for ALD.

Numerous studies have examined the role of miRNAs in different stages of ALD using both humans and animal models (see Appendix 2E). miR-30e,^86^ miR-192, miR-122, and miR30a,^87^ and miR-182^88^ were dysregulated in AH and were related to the progression and severity of AH. miR-21 regulated the nuclear factor kappa-B (NF-kappa-B) pathway, thereby playing a role in modulating hepatic inflammation and reducing inflammatory cytokine release during ALD.^89^ In people with ASH, an increase in miR-432 and sodium-coupled neutral amino acid transporter 1 (SLC38A1) gene expression in the liver correlated with poor survival outcomes, suggesting a potential role of miR-432 in the pathogenesis of ASH.^90^ Furthermore, ethanol exposure decreased miR-192-5p in ASH, which negatively regulated expression of fibronectin type III domain containing 3B (FNDC3B), leading to inactivation of adenosine monophosphate-activated protein kinase (AMPK) and contributing to iron overload and iron-dependent cell death (ferroptosis).^91^ A common feature in alcohol-associated fatty liver disease is a dysfunction in lipid metabolism. miR-181b-5p was reported to target protein arginine N-methyltransferase 1 (PRMT1), which is associated with various cellular processes, and miR-378b was shown to target calcium/calmodulin-dependent protein kinase kinase 2 (CaMKK2), which is involved in activating AMPK, to influence lipid metabolism.^92^,^93^

Other studies examined the role of miRNAs and their molecular mechanisms in alcohol-induced liver injury and liver fibrosis in human and mouse samples (see Appendix 2E). The studies found that miR-122 and miR-214 were consistently altered in response to alcohol, affecting antioxidant enzyme levels and fibrotic gene expression.^94^–^98^ Other miRNAs such as miR-145 miR-34a also were implicated as inducers of fibrosis via transforming growth factor-beta (TGF-beta)/SMAD signaling and modulation of macrophage polarization, respectively,^99^–^101^ underscoring the relevance of miRNAs as potential targets for therapeutic interventions in ALD.

#### Alcohol use disorder/alcohol dependence

Long-term use of alcohol may induce changes in miRNA expression. Studies have identified altered miRNA expression associated with AUD or alcohol dependence across various species and sample types (see Appendix 3A). Animal models have contributed significantly to our knowledge of key biological mechanisms mediated by miRNAs. For instance, the earliest animal study in this area linked miR-9 to the development of acute tolerance to alcohol, which resulted from the miRNA’s ability to change the balance of transcripts with alternate 3′UTRs for the BK potassium-activated sodium channel.^102^ In other animal models, key miRNAs, such as miR-30a-5p and miR-206-3p, were upregulated,^103^ with miR-206 playing a role in increased alcohol self-administration and reduction of brain-derived neurotropic factor (BDNF) expression.^104^ However, miR-30a-5p restored BDNF levels and decreased alcohol consumption.^105^ In humans, differential expression of miR-92, miR-122, and miR-146 also has been observed, with miR-92 and miR-122 generally downregulated in people with AUD^106^–^108^ and miR-146 upregulated,^106^,^109^,^110^ particularly in persons classified as heavy drinkers (i.e., who reported two or more instances of binge drinking or consumed the equivalent of ≥1.0 oz. of pure ethanol per day^68^). Another study showed that miR-124-3p was increased in the nucleus accumbens and decreased in the limbic forebrain after ethanol withdrawal in animal models of alcohol dependence.^111^ Only one study found sex differences in differential expression of miRNAs after ethanol exposure; for example, miR-125a-3p and let-7a-5p were upregulated in males, but not females, whereas miR-881-3p and miR-504 were downregulated in females, but not in males.^112^ A number of mechanistic studies also have assessed behavioral endpoints. For instance, inhibition of miR-137^113^ and overexpression of let7d^114^ have been shown to reduce alcohol consumption, indicating their potential as therapeutic targets.

Several miRNAs associated with different stages of AUD (i.e., alcohol consumption, dependence, and withdrawal) are known to regulate mRNA networks involved in neurotransmission, neuroadaptation, and synaptic plasticity.^115^ For example, miR-130a targets mRNAs for the inositol 1,4,5triphosphate receptor type 2 (ITPR2) and the Na^+^/K^+^ transporter ATP1A2, which are involved in ion channel regulation.^116^ Genetic variations in miRNA genes may also contribute to alcohol misuse. For instance, a G>A polymorphism (rs2910164) in miR-146a was significantly more prevalent in people with AUD compared to sex-matched people without AUD, indicating a genetic predisposition linked to miRNA regulation.^117^ In another study, the level of gamma-aminobutyric acid receptor delta (GABAAR-delta) protein in the dorsal hippocampus was inversely related with miR-365-3p in high-alcohol preferring mice, suggesting genetic background can contribute to miRNA-mediated post-transcriptional mechanisms.^118^

These studies highlight the multifaceted role of miRNAs in AUD as they influence various neurobiological processes and offer potential avenues for diagnostic and therapeutic interventions. Some of these studies have used saliva samples to assess the association between miRNAs and AUD,^106^,^119^ which suggests the possibility of developing point-of-care devices for conveniently assessing AUD risk and severity. However, further research is necessary to explore and validate these current findings.

#### Neurological inflammation

Appendix 3B summarizes studies assessing the regulatory roles of miRNAs in alcohol-related neuroinflammation, neurotoxicity, and cellular stress responses. These studies used samples ranging from blood to various brain regions in species ranging from mice to macaques and humans, and employed a variety of analytic techniques. miR-155 was frequently upregulated in response to chronic ethanol feeding in rats and mice, contributing to neuroinflammation and inflammatory cytokine production via toll-like receptor 4 (TLR4) and TLR7 signaling pathways.^120^,^121^ Other studies using mouse models such as TLR4 knockout mice also highlighted alterations in miRNAs that modulate TLR4 and NF-kappa-B pathways following ethanol exposure.^122^–^125^ miR-155 is a pro-inflammatory mediator of the central nervous system and is upregulated in the brain of people affected by many neurodegenerative diseases.^126^,^127^ It may also modulate numerous mechanisms contributing to the etiology of Alzheimer’s disease.^128^ The induction of neuroinflammatory miRNAs following ethanol exposure during development and in adults offers potential targets for therapeutic interventions in alcohol-related neurobiology.

#### FASD/neurodevelopmental disorders

Research has evaluated the impact of ethanol exposure on miRNA expression across various species, tissues, and developmental stages (see Appendix 3C). Using diverse systems, from zebrafish and mice to sheep and humans, and sample types ranging from blood to embryonic tissues, and diverse analytic techniques, studies have shown that ethanol exposure significantly alters miRNA expression, impacting a variety of biological processes.

Several miRNAs, including miR-9, miR-15b, miR-19b, and miR-20a, were dysregulated in ovine blood upon ethanol exposure,^129^ indicating they could be candidate biomarkers for maternal ethanol exposure, as well as biomarkers in newborns to test for fetal exposure. In human embryonic stem cells, miR-145 mediated alcohol toxicity by targeting the transcription factor Sox-2 and extracellular signal-regulated kinases, resulting in neural progenitor depletion.^130^ In another study, the signaling cascade involving the transcription factor SP1, protein kinase R (PKR), and PKR-associated protein X (RAX) was linked to ethanol-induced cerebellar neuron apoptosis via miR-29b in mice.^131^

miR-9, the miRNA most often identified in studies of neurological and developmental effects (see Appendix 3), is important for neural stem cell maturation and brain development.^132^,^133^ Multiple studies across different species and tissues, including zebrafish embryos,^134^ murine blood,^135^ ovine blood,^129^ monkey brain,^136^ and human brain and fetal central nervous system-derived extracellular vesicles^137^ have shown dysregulation of miR-9 upon ethanol exposure, highlighting its significance in these processes. These findings highlight the complex interplay between ethanol exposure and miRNA regulation, underscoring the potential of miRNAs such as miR-9 as biomarkers for ethanol-induced developmental disorders and as therapeutic targets for mitigating the adverse effects of prenatal alcohol exposure.

#### Knowledge gaps: miRNA regulation and miRNA effects

A major focus of studies of miRNAs in the context of AUD and associated conditions has been on screening tissues to identify and enumerate the miRNAs that are responsive to alcohol exposure in brain tissues and in organs that are secondary AUD targets (see Table 3). Relatively few studies have focused on mechanisms that link ethanol to changes in miRNAs. Some linking mechanisms that have been identified include methylation changes in miRNA gene loci^43^,^138^ and promoter occupancy by chromatin remodeling factors such as the BRG1/BRM-Associated Factor (Baf) complex.^139^ In general, however, this area has been poorly investigated.

Similarly, gaps remain in understanding the effects of miRNAs, whether acting alone or in groups of co-regulated miRNAs. Most studies on the roles of miRNAs in AUD have focused on single miRNAs. For instance, an early study showed that striatal explant cultures obtained from juvenile male rats and treated with ethanol exhibited an increase in miR-9 and a loss of splice variant transcripts of the BK potassium channel whose 3′UTR contained miR-9 binding sites.^102^ The authors of that study interpreted this as a mechanism for ethanol tolerance in striatal neurons, an important phenotype for the emergence of AUD. A subsequent study implicated another important brain-expressed miRNA, miR-124a, and its regulated target, BDNF, in alcohol preference.^140^ Elevation of miR-124a levels by microinjection into the dorsal striatum of adult male rats resulted in decreased BDNF levels and increased alcohol preference in a conditioned place preference and two-bottle choice paradigm; conversely, miR-124a inhibition resulted in decreased voluntary alcohol consumption.^140^ Importantly, these studies only included male rats; therefore, it is unknown if similar miRNA mechanisms facilitate alcohol tolerance, preference, and consumption in females. The lack of studies powered to detect differences between males and females represents a significant limitation to early studies that is only recently being addressed.

These early studies also did not specify whether for the miRNA in question the guide or the passenger strand of the miRNA strand was retained following DICER cleavage. It may be reasonable to assume that the studies implicated the dominant (guide) strand of the miRNA (i.e., miR-9-5p and miR-124a-5p). However, caution is warranted, because passenger strand miRNAs (e.g., miR-9-3p) also can be retained,^141^ be differentially regulated by ethanol,^142^ and exhibit a unique functional specificity.^143^

According to the dominant conceptual model, miRNAs exert their influence by binding to the 3′UTRs of target mRNAs to destabilize the mRNAs and thereby inhibit gene translation. A number of studies utilized the approach of creating reporter constructs in which the luciferase gene (whose activity can easily be measured) was linked to the 3′UTR of the presumptive target mRNA to explore the mechanistic link between ethanol-sensitive miRNA and mRNA translation.^97^,^102^,^144^–^146^

Early studies already noted the potential for miRNAs to cooperatively target mRNA translation.^35^ Most miRNA-regulated mRNAs have multiple miRNA target sequences within their 3′UTRs, and most miRNAs target multiple mRNA 3′UTRs. Therefore, the potential for cross-talk between miRNAs is an important but poorly studied component of AUD biology. An early study did present evidence that miRNAs inhibited by ethanol in neural progenitor cells cooperatively controlled cell survival and target gene expression.^41^ A more recent study identified a cluster of 11 miRNAs that are elevated in the second trimester of pregnancy, in the plasma of some alcohol-exposed pregnant women. These miRNAs collectively explained 24%–30% of the variance in growth metrics in newborn infants, collectively inhibited placental growth in mice, and inhibited invasiveness of human trophoblast cells.^147^ However, the contribution of differentially regulated miRNA clusters to the pathogenesis of AUD has rarely been investigated to date.

#### Other sources of miRNA variation

RNA sequencing studies have shown that individual miRNAs within a single organism can exhibit substantial sequence heterogeneity due to processing variations and post-transcriptional modifications. DICER processing of a given pre-miRNA can be imprecise and result in a cell-type–dependent heterogeneity of mature miRNA products,^148^ for example, in the 5′-seed sequence for targeting mRNAs. Nucleotide polymorphisms within miRNA genes can also contribute to variation in the size and sequence of mature miRNAs.

Another cause of miRNA variation are adenosine deaminases of RNAs (ADARs), a class of enzymes that can change adenosine to inosine (A-to-I editing) in RNAs, including miRNAs. A-to-I editing of pri-miRNAs can block cleavage by DICER.^149^ However, ADARs can also form a complex with DICER to facilitate miRNA processing; conversely, loss of ADARs can globally inhibit miRNA expression.^150^ A-to-I editing within a miRNA seed sequence can alter targeting specificity and function.^151^ Moreover, A-to-I editing within mRNA transcripts can also reveal new miRNA regulatory sites,^152^ suggesting that mechanisms that control RNA editing can also result in dynamic changes in miRNA:mRNA interactions. RNA editing of protein-coding genes has been shown to regulate alcohol consumption,^153^ but the effects of alcohol on editing miRNAs are unknown.

Like lncRNAs (discussed below) and mRNAs, miRNAs can also be post-transcriptionally modified by methylation. For instance, some miRNAs that are associated with AUD targets (e.g., miR-17-5p, miR-21-5p, miR-200c-3p, and let-7a-5p) exhibit increased methylation in cancer.^154^ A recent paper provided evidence that members of the let-7 miRNA family were subject to 7-methylguanosine (m7G) methylation by the enzyme METTL1 and that m7G methylation of pri-Let-7e promoted more efficient processing of pri-miRNA to pre-miRNA by Drosha.^155^ 6-methyladenosine (m6A) methylation has also been described in miRNAs and shown to be important for promoting pri-miRNA processing.^156^ Numerous studies have shown that ethanol both increases and decreases a large number of miRNAs and that at least some of the regulation may occur at the level of methylation. However, no studies to date seem to have identified ethanol effects on the METTL RNA methyltransferase family, although one study noted that operant ethanol self-administration resulted in significant elevation of tRNA methyltransferase 1 mRNA transcripts in prefrontal cortex of young adult male rats.^157^ Although the study included no females, representing a common gap in our knowledge, these data support a more in-depth analysis of miRNA methylation machinery in AUD pathology.

#### Other sources of RISC complex variation

As mentioned above, the activities of RISCs can be modulated by ADARS. A large number of RISC binding partners have been identified, suggesting that the association between argonautes and their binding partners can contextualize miRNA control over cellular translation. The RNA-binding protein fragile X messenger ribonucleoprotein 1 (Fmr1) is an example of the potential intersection between RISC function and AUD pathology. Fmr1 by itself can promote Ago activity; however, when it binds to the RISC-associated helicase, Mov10, it inhibits the translational-repression activity of Ago proteins.^158^ Fmr1 phosphorylation promotes the translation-inhibitory activity of Ago, while Fmr1 dephosphorylation associated with cell-surface G-protein-coupled-receptor (GPCR) signaling results in increased protein translation.^159^ This implies that GPCR signaling at a synapse can switch on local protein synthesis by controlling the phosphorylation state of an Ago-associated protein. The contextual environment of a RISC complex member like Fmr1—that is, phosphorylation state and binding partner availability—is important because a number of studies have linked ethanol consumption with elevations in brain Fmr1.^160^–^162^ These studies have also linked elevations in Fmr1 with shifts in glutamate and GABA plasticity, advancing a potential miRNA-mediated mechanism in AUDs. It will be important for future studies to directly link RISC proteins and their activation states with synaptic plasticity responses to ethanol among others.

#### miRNA compartmentalization

miRNAs can be found in different parts of the cell or can be secreted from cells. The dominant conceptual model is that miRNAs are processed and act in the cytoplasm of cells and tissues to inhibit the translation of their target mRNAs. Indeed, most of the reviewed studies used this conceptual framework. However, once miRNAs have been processed in the cytoplasm, they can be translocated back to the nucleus. Evidence in support of nuclear functions of miRNAs comes from studies showing that Ago proteins (which act as chaperones of RISC complex and thus miRNAs) can bind the protein trinucleotide repeat containing adaptor 6A (TNRC6A), which has both nuclear localization and export signals and can shuttle between nucleus and cytoplasm.^163^ Mature miRNAs also have been shown to translocate to the nucleus, where they can regulate transcription. For instance, Ago1 protein was found in the promotor region of the mouse cyclin B1 (Ccnb1) gene, and the miRNA miR-744, which targets a site in the promoter of the Ccnb1 gene, increased RNA polymerase-2 occupancy at the transcription start site, Ccnb1 transcription, and cell proliferation.^164^ Several publications subsequently documented both transcription activation and transcription silencing due to nuclear miRNA and RISC complex activity (for a review, see Bhattacharjee et al. 2019^165^). One recent study linked alcohol exposure to nuclear actions of miRNAs.^166^ The authors showed that in a hepatocellular carcinoma cell line, ethanol exposure resulted in decreased levels of miR-29c, a miRNA that was present in the nucleus and exhibited Ago2-dependent binding to the enhancer region of the alcohol dehydrogenase 6 (ADH6) gene, but not to the 3′UTR of ADH6 mRNA. In their model, miR29c activated the enhancer and recruited RNA polymerase II to ADH6 and other ADH genes to promote gene transcription. These data provided evidence that a miRNA acting within the nucleus could mediate epigenetic effects of ethanol. Given the link between alcohol and cancer, such transcription control may represent an important mechanism for tumor progression that warrants further investigation.

Other studies showed that miRNAs could also be secreted in biofluids and serve as biomarkers for disease.^167^–^169^ Subsequently, evidence emerged that secreted miRNAs were chaperoned by Ago proteins,^170^ lipoproteins such as high-density lipoprotein, 171 or in small extracellular vesicles.^172^ AUD-related studies showed that miRNAs whose levels were elevated in plasma extracellular microvesicles could serve as biomarkers for ALD in mice.^73^ Moreover, in an ovine model of pregnancy, maternal circulating miRNAs were biomarkers for fetal alcohol exposure.^129^

Circulating miRNA research accounts for about 18% of the publications on miRNAs in AUDs identified in this scoping review. To date, most published papers have reported on screening circulating miRNAs as biomarkers for prenatal alcohol exposure and associated pathology. Only a few studies have used circulating miRNAs in predictive models for future disease burden due to alcohol. For example, two papers showed that a cluster of microRNAs that was elevated in blood plasma of pregnant women during the second trimester predicted growth deficits of newborn infants.^129^,^147^ A transition in the field from enumerating the effects of alcohol and AUD pathology on circulating miRNA expression to a focus on predicting future health outcomes associated with alcohol exposure, including future disease burden and mortality from miRNA profiles, is likely to yield substantial gains in health management for AUDs.

Circulating miRNAs also have the potential to serve as endocrine molecules—that is, when they are secreted into circulation by cells and tissues, they can affect the biology of recipient cells and tissues. Some studies have tried to assess the endocrine potential of secreted miRNAs. This has been useful and informative when the focus has been on individual miRNAs. For example, one study showed that alcohol exposure resulted in increased release of miR-122 from hepatocytes in extracellular microvesicles, and that recipient monocytes exhibited inhibition of the heme oxygenase 1 pathway and increased sensitization to lipopolysaccharide stimulation.^173^ A second study showed that miR-27a release from alcohol-treated monocytes into extracellular vesicles resulted in increased expression of the mannose receptor CD206 and the hemoglobin-haptoglobin scavenger receptor CD163, as well as transformation of naïve monocytes into M2-type macrophages.^174^ Such macrophages may be mediators of fibrosis and and/or tumorigenesis in tissues such as liver.^175^ A third paper showed that ethanol exposure resulted in increased miR-140-3p levels in extracellular microvesicles secreted by cultured mouse neural progenitor cells and that a molecule mimicking miR-140-30 (miR-140-3p mimetic) increased cell proliferation of neural progenitors.^176^ Finally, one study presented a novel, atypical mechanism for secreted miRNA activation of an inflammatory response, resulting in a transfer of Let-7b from Ago to the proinflammatory high mobility group box 1 protein in microvessels, for presentation to TLR7.^177^ This mechanism was associated with subsequent neurotoxicity. This last study emphasizes the diversity of chaperone–miRNA interactions that may be activated by ethanol.

These types of studies on individual miRNAs are valuable but have limited impact for understanding AUD pathology, because effects of ethanol are often associated with changes in the extracellular release profile of a large number of miRNAs. The collective action of this miRNA cohort on recipient tissues may not reflect the behavior of their individual component miRNAs. Some studies have attempted to make predictions about endocrine biology of secreted miRNA cohorts using pathway-enrichment analysis (e.g., see Jing et al. 2015^178^). However, such an approach provides, at best, a theoretical framework that needs to be tested experimentally. Two studies did attempt a comprehensive analysis of the cohort behavior of secreted miRNAs.^147^,^179^ Based on data showing that 11 maternal circulating miRNAs that were elevated in the second trimester of pregnancy predicted infant birth outcomes following prenatal alcohol exposure,^180^ these studies assessed the behavior of individual and grouped miRNAs on placental trophoblast gene expression, trophoblast behavior, and fetal growth. One study found that as a group, the 11 miRNAs inhibited epithelial-to-mesenchymal transition (EMT) genes in human trophoblast cells, inhibited trophoblast invasion, and explained the effects of prenatal ethanol on EMT in rodent and primate models.^147^ Moreover, injecting eight out of these 11 maternally elevated miRNAs into a naïve pregnant mouse at mid gestation resulted in fetal growth restriction compared to controls, a hallmark phenotype of prenatal alcohol exposure. Importantly, the behavior of no single secreted miRNA predicted the behavior of the miRNA cohort. A subsequent transcriptomic study showed that this cohort of miRNAs collectively upregulated Notch signaling pathway genes, dysregulated angiogenic genes in placenta, and decreased umbilical cord blood flow.^179^ Collectively, these data emphasize the functional, endocrine nature of miRNAs that are secreted into biological fluids in conjunction with AUD pathology. The translational potential of these findings is strong, suggesting that miRNA mimetics or antagonists may be delivered into circulation to mitigate effects of alcohol exposure.

#### Sex as a biological variable in miRNA studies

Only a minority of miRNA studies (approximately 39%) reported using female subjects, and it is not clear if the studies were adequately powered to detect sex differences in miRNA regulation or miRNA-dependent biology. Newer statistical methodologies, such as bootstrap-resampling approaches— which involve repeatedly sampling from the data with replacement to estimate the accuracy and variability of statistical measures—can help overcome false discovery rate barriers. These methods can be applied in secondary data analyses to identify the contributions of important biological variables, such as genetic sex, age, and others. For instance, a secondary analysis of maternal miRNA profiles in pregnancy using such a bootstrap resampling approach uncovered distinct maternal circulating miRNA profiles in pregnancies with male and female fetuses.^181^

### Long Noncoding RNAs (lncRNAs)

lncRNAs have been arbitrarily defined as RNAs that exceed 200 nucleotides in length, but many exceed several kilo bases (kb) in length. For instance, lncRNA kcnq1ot1/NONCODE transcript ID# NONHSAT017523.2, which is encoded on the antisense strand for the protein-coding gene kcnq1, is estimated to exceed 91kb in length.^182^ The human genome is annotated to encode more than 172,000 lncRNA transcripts, while the mouse genome encodes more than 131,000 lncRNA transcripts.^182^ However, most lncRNAs are not well-characterized, and the functions of many are simply unknown. While a large number of lncRNAs are conserved, many others are specific to humans, with functions that are not present in other species. This means that lncRNA biology relevant to human alcohol use and to AUD may be both evolutionarily conserved and human-specific. Compared with miRNA research, little work has been done on lncRNAs and alcohol. Approximately 10% of the assessed papers in this review dealt with lncRNAs. Especially given the large number of annotated lncRNAs, the published literature is sparse and presents a limited window on their contribution to the biology of AUD and their effects. Appendix 4 summarizes the methods and key findings of various studies examining lncRNAs in the context of alcohol-related diseases.

#### Modes of lncRNA action: Nuclear functions

lncRNAs are generally thought to play a critical role in the packaging and modification of the nuclear chromatin structure for epigenetic regulation of gene expression, but may have a range of additional and mostly unexplored functions (for a consensus statement, see Mattick et al. 2023^10^). In humans, lncRNA UBE3A-ATS (SNHG14), which is encoded on the antisense strand for the gene encoding the ubiquitin ligase UBE3A,^183^ is an important example of functional epigenetic control. This lncRNA, encoded from the paternal allele in the nervous system, epigenetically silences the paternal UBE3A allele; therefore, loss of the maternal UBE3A allele (e.g., due to mutation or deletion) can result in developmental disability (Angelman Syndrome).^184^ Studies with other gene-silencing lncRNAs, such as XIST, have shown that gene silencing is mediated by the presence of repeating stem-loop structures, whereas chromatin binding is mediated by other portions of this lncRNA.^185^ Moreover, the organization of targeted chromatin structure can also influence the efficacy of lncRNAs. For instance, regions of chromatin that have a low density of long interspersed nuclear element-1 repeats are relatively resistant to XIST-mediated silencing.^186^ These data suggest that sequence and structure of lncRNAs as well as the structure of target chromatin may be important determinants of epigenetic regulation by lncRNAs. Finally, epigenetic control mechanisms mediated by a single lncRNA may be dependent on cell-types and physiological context. For instance, cell-types such as B-lymphocytes, which are subject to substantial genetic recombination to generate antibody diversity, utilize XIST in a unique complex of proteins to stall translation of specific X chromosome-linked immune genes such as TLR7 that do not contain sequences (i.e., CpG islands) susceptible to DNA methylation.^187^ Such cell-contextual partnering between lncRNAs and epigenetic machinery has not been explored in the context of AUD and related effects.

Like UBE3A-ATS, numerous lncRNAs are transcribed from the DNA strand opposite to that which encodes a protein; they are therefore referred to as long antisense RNAs, with a nomenclature that acknowledges the antisense position relative to the protein-coding gene. BDNF-AS, the lncRNA encoded on the opposite strand to the gene coding for BDNF. Decreased BDNF in the dorsolateral striatum itself resulted in increased alcohol self-administration in a rat model.^188^ However, BDNFAS, which acts as an inhibitor of the BDNF,^189^ was found to be elevated post-mortem in the amygdala of persons with a diagnosis of early-onset AUD; additionally, BDNF-AS recruited the methyltransferase protein enhancer of zeste homolog 2 (EZH2) to the BDNF promoter, resulting in promoter silencing.^190^ Antisense lncRNA transcripts are associated with many important growth and plasticity genes, and their actions, as with the BDNF/BDNF-AS gene may mediate many pathogenic effects of ethanol, at the genomic level, but little is known about how such pairs of genes function.

Aside from epigenetic mechanisms, lncRNAs can simultaneously exhibit multiple alternate nuclear functions. The abundant nuclear lncRNA, metastasis associated lung adenocarcinoma transcript 1 (MALAT1), also known as nuclear enriched abundant transcript 2 (NEAT2), was upregulated in postmortem brain samples from people with a diagnosis of AUD and increased in rat brain following withdrawal from ethanol.^191^ It was associated with “nuclear speckles”^192^—sites of active mRNA transcription.^193^ MALAT1/NEAT2’s partner in the nucleus, NEAT1, which is elevated in ALD,^194^ is a pertinent example alternate isoforms of individual lncRNAs having different functions. For instance, NEAT1 exists in two isoforms of 23kb and 3.7kb length. The 23kb isoform normally localizes within the nucleus where it serves as an architectural RNA for the assembly of paraspeckle bodies^195^ (i.e., interchromatin structures adjacent to MALAT1/NEAT2-enriched nuclear speckles) that may be important for retaining double-stranded RNA that has been subject to A-to-I editing.^196^ However, NEAT1 can be subject to alternate 3′-polyadenylation, and the 3.7kb polyadenylated isoform does not retain the capacity to assemble paraspeckle bodies;^197^ instead, it may inhibit paraspeckle body formation and have alternate, perhaps antagonistic functions in cells.^198^,^199^ Alternate polyadenylation and splicing variations in lncRNAs have not been investigated in AUD pathology.

Post-mortem analysis of brain tissues documented that a large number of lncRNAs, including long antisense RNAs and pseudogene transcripts (RNAs transcribed from presumably non-functional gene copies of protein-coding genes) were either elevated or suppressed coincident with a diagnosis of AUDs.^200^ The authors of that study used gene ontology—an analytic approach for systematic and consistent classification of gene functions, regardless of the organism—to advance a hypothesis that the dysregulation of these lncRNAs was associated with genome-wide mis-splicing of RNA transcripts. This hypothesis is an innovative and testable link between lncRNAs and alcohol’s pathogenic effects, but it is unlikely to be the only or even major mechanism of alcohol-related pathogenesis. Increasing evidence suggests that most, if not all, lncRNAs have multiple, context-dependent functions, and each lncRNA needs substantial further investigation.

#### Modes of lncRNA action: Cytoplasmic functions

Changes in location of ncRNAs within cells (i.e., translocation between nucleus and cytoplasm) can dramatically alter the function of an ncRNA. For instance, under stress or inflammatory conditions, NEAT1 in macrophages can also localize to the cytoplasm and, for example, stabilize caspase-1 enzyme and promote the production of interleukin-1b.^201^ It will be important to analyze how cytoplasmic lncRNAs interact with their protein partners in AUDs—for example, using RNA-immunoprecipitation methodologies.^202^

Within the cytoplasm, NEAT1 also functions as a “competing endogenous RNA” (ceRNA), serving as a “sponge” to sequester miRNAs.^203^,^204^ In a study of ASH, Ye and colleagues showed that levels of NEAT1 and miR-129-5p were inversely related to each other and that the lncRNA/miRNA pair behaved as functional antagonists, which suggests a ceRNA function for NEAT1.^194^ Its nuclear partner, MALAT1/NEAT2, can also serve as a miRNA sponge.^205^ Similarly, BDNF-AS lncRNA, which was linked to epigenetic mechanisms, also exhibits miRNA sponge activity by sequestering the miR-9-5p,^206^ which has been implicated in animal models in the pathogenesis of both fetal effects of ethanol^41^,^139^,^207^,^208^ and of AUD.^102^ BDNF-AS therefore classifies as a ceRNA, although its potential ceRNA activity in AUD and related consequences has not been explored. Another antisense RNA, HOX transcript antisense RNA (HOTAIR), has been linked to pathogenic effects of ethanol in bone and liver^209^–^211^ and has been shown to exhibit miRNA sponge activity in its contribution to ethanol-related pathogenesis.^209^,^210^ Other antisense strand-encoded lncRNAs may exhibit similar ceRNA activity in the context of ethanol effects and need to be further investigated.

Potential ceRNA activity for lncRNAs can include not only miRNA sponge activity, but also the direct control of gene translation via regulation at the 3′UTRs of mRNAs. An example is a lncRNA encoded from a pseudogene locus (Oct4pg9), which was created by duplication of the Oct4/Pou5f1 gene (a core transcription factor for stem cell identity). In developing cortical neural progenitor cells, Oct4pg9 was elevated following ethanol exposure.^212^ This study showed that Oct4pg9 lncRNA not only binds miRNAs and their Ago chaperones (i.e., has a sponge activity) but also directly and independently inhibited translation of a reporter gene linked to the Oct4/Pou5f1 3′UTR. This suggested a novel ceRNA mechanism whereby Oct4pg9 lncRNA directly repressed translation of Oct4/Pou5f1,^213^ providing evidence that the paired protein-coding gene and pseudogene-encoded lncRNA could mediate loss of stem cell identity following ethanol exposure. In general, the ceRNA function of lncRNAs warrants substantial further investigation.

#### Modes of lncRNA action: Endocrine/paracrine lncRNAs

lncRNAs, like miRNAs, also are secreted by cells into extracellular spaces and fluids, where they may serve as endocrine molecules. For instance, studies documented that NEAT1 is secreted in extracellular vesicles and can regulate pathogenic processes, including cardiac fibrosis^214^ and cancer^215^ by exploiting its various modes of function in recipient target cells. The role of endocrine lncRNAs in alcohol pathology is largely unknown. Moreover, the presence of lncRNAs in body fluids such as blood presents an exciting opportunity to assess these molecules for diagnostic purposes. Some published studies have indeed assessed lncRNAs in biological fluids as markers for ALD^194^,^216^,^217^ and esophageal cancer,^218^ and these molecules could be further investigated as biomarkers for other AUD-associated diseases.

#### Modes of lncRNA action: Posttranscriptional processing

Following transcription, all RNAs may be modified by the addition of methyl groups, mainly to adenosines (m6A^219^), although methylation at cytosines and guanosines (m5C, m7G, etc.) can also occur. Such epi-transcriptomic modifications can control a variety of processes, including RNA splicing, export, stability, and translation.^220^ RNA can also be more directly modified by direct editing of base sequences, such as A-to-I editing by ADARS,^221^ resulting in alterations in the information content of an RNA sequence. Both types of post-transcriptional modifications have been described for lncRNAs as well,^222^,^223^ but remain a poorly investigated area of study in general, and in AUDs specifically.

In cancer cell-culture models, ethanol exposure has been shown to reduce m6A methylation around the stop codons for opioid receptor mRNAs.^224^ In human populations, mutation patterns and expression of genes regulating m6A mRNA methylation predicted disease-free intervals in patients with alcohol-associated hepatic carcinoma,^225^ supporting the hypothesis that RNA methylation mechanisms may mediate pathogenic effects of ethanol. However, the role of such epi-transcriptomic modifications of lncRNAs that mediate ethanol effects remains to be determined.

Other studies in animal models found that editing of protein-coding mRNAs, such as the brain 5HT2C serotonin receptor, increased as a consequence of ethanol exposure.^153^,^226^ Moreover, inhibiting A-to-I editing of serotonin receptor in the nucleus accumbens decreased alcohol intake after chronic ethanol exposure,^153^ suggesting that A-to-I editing has functional consequences for both molecular and behavioral manifestations of how alcohol impacts the individual (i.e., AUD phenotypes). However, the effects of ethanol on lncRNA editing or the consequences of such editing remain to be studied.

lncRNAs can also be post-transcriptionally processed to generate small RNAs with divergent functions. The 3′-end of the ethanol-induced nuclear MALAT1/NEAT2 lncRNA can be cleaved by RNAse-P to generate a small tRNA-like fragment, MALAT1-associated small cytoplasmic RNA (mascRNA), that, unlike its parent lncRNA, localizes to the cytoplasm. MascRNA was found to globally promote gene translation by stabilizing a component of the multi-tRNA synthase complex.^227^ However, mascRNA can also promote protein ubiquitination and proteasomal degradation,^228^ suggesting a more nuanced role for this small RNA as a mechanism to control protein turnover in cells. It remains to be determined whether elevated levels of MALAT1/NEAT in AUD imply a commensurate increase in mascRNA. However, a role for mascRNA in AUD pathology is suggested by findings that mascRNA modulates effects of TLRs, inhibiting downstream activation of TLR4 but facilitating TLR3.^228^ Both TLR3 and TLR4 have been shown to be increased in rat brain following ethanol exposure and mediate inflammatory responses in these models.^229^,^230^ Thus, ncRNAs such as mascRNA may add nuance to AUD-associated inflammation by shaping the balance between inflammatory pathways.

#### Modes of lncRNA action: Micro-peptide translation

lncRNAs have been labelled as non-protein–coding RNAs because they do not appear to possess conventional protein-encoding open reading frames. However, uncertainty persists around whether lncRNAs truly are non-protein-coding or could be translated under some conditions. For example, early evidence suggested that lncRNAs could associate with ribosomes, suggesting that they may in fact be translated.^231^ A 2014 study examining the structure of lncRNAs suggested that these RNAs may indeed contain short open reading frames that may encode small peptides (micropeptides).^232^ Finally, in 2015, researchers reported that a micropeptide termed myoregulin was indeed encoded by a lncRNA (human annotation: LINC00948, mouse: 2310015B20Rik), was expressed in skeletal muscle, and regulated Ca^2+^ uptake into the sarcoplasmic reticulum.^233^ Since that report, several publications have documented that, aside from their non-protein-coding functions, lncRNAs do contain short open reading frames and can simultaneously encode micropeptides.^234^–^236^ Moreover, pri-miRNAs, a class of lncRNAs that have hitherto been solely regarded as precursors for miRNAs, also may simultaneously encode micropeptides with independent functions,^237^ suggesting a new level of biological complexity associated with lncRNAs that parallels the complexity of miRNA biology. The emerging data suggest that the activity of lncRNAs may be contextual and result, at times, in these RNAs serving as templates for translating small peptides.

This contextuality of lncRNA biology presents an exciting area of research that has not been explored in the field of AUD biology.

#### lncRNAs mediate sex-specific effects of ethanol

Genetic sex is increasingly identified as a key contributor to individual differences in disease susceptibility; however, until recently, sex differences in AUD have been poorly documented (for a review, see Becker and Koob 2016^238^). This deficiency also exists for the emerging knowledge base of lncRNA contributions to biology. For instance, the first two lncRNAs to be identified— H19, a paternally imprinted lncRNA^14^,^239^ associated with developmental growth control,^240^ and XIST, the female-specific lncRNA^13^,^241^ responsible for inactivating the supernumerary X-chromosome in female cells as a dose compensation mechanism^242^—are also targets of ethanol, both during development and in the adult, but with sex-specific differences. For example, prenatal alcohol exposure in a mouse model increased brain H19 lncRNA, but only in male offspring.^243^ It will be important to identify a functionally equivalent lncRNA in female offspring. Indeed, when lncRNAs are regulated or act in a sex-specific manner, it will be important to identify equivalent biological mechanisms that contribute to common outcomes. Increased H19 levels were shown to promote processing of another lncRNA, Homer, into a stable, covalently closed loop (i.e., circularization) leading to higher levels of circHomer (for more on circRNAs, see below). The potential circHomer-specific effects were observed only in male offspring.^243^ It remains unknown whether equivalent regulatory mechanisms contribute to alcohol’s effects in females, highlighting an important area for future research.

The involvement of XIST as a mediator of alcohol effects, in contrast, suggests the existence of female-specific mechanisms. XIST is a female-specific lncRNA that randomly and epigenetically silences one of two X chromosomes (for review, see Brockdorff et al. 2020^244^), resulting in chromatin condensation and Barr body^245^ formation. Three papers have connected ethanol exposure to XIST. A study in human liver stellate cells showed that ethanol increased levels of XIST,^246^ while another study in mouse neuroblastoma cells found that ethanol exposure transiently increased and then decreased XIST levels.^247^ These two papers present a partly overlapping outcome resulting from ethanol exposure. Finally, a third study, using a single-cell RNA-seq approach, showed that a single episode of ethanol exposure in a pregnant mouse at the onset of fetal neurogenesis resulted in widespread decreases in expression of XIST across multiple cell types in the developing female fetal cerebral cortex, with evidence of loss of X chromosome inactivation (i.e., increased transcript abundance from X chromosome-encoded genes).^248^ The partial discrepancy between studies highlights the fact that more research is needed to define the contributory role of intervening factors such as variations in the sensitivity of various cell/tissue types to ethanol, and the effects of alcohol exposure pattern (e.g., episodic/acute vs. chronic) and dose on outcomes. Moreover, since XIST is a female-specific transcript, it will be important to determine whether equivalent, but XIST-independent, mechanisms exist in males. At a more global level, a greater understanding of the sex-specific roles of lncRNAs will pave the way for precision medicine approaches to treating AUD.

### Circular RNAs

circRNAs are a type of ncRNA with a looped structure without 5′ or 3′ ends. This structure makes them more stable than linear RNAs due to their resistance to exonuclease. circRNAs are generated from precursor mRNAs (pre-mRNAs) through back-splicing of exons (exonic circRNAs), introns (circular intron RNA), or both (exon-intron circRNAs).^249^ Among these, exonic circRNAs are the most abundant type. There are three main models for the biogenesis of circRNA: lariat-driven, intron-pairing-driven, and RNA-binding proteins (RBPs)-driven circularization. In the lariat-driven circularization, the pre-mRNA forms a loop (lariat) intermediate that contains introns and/or exons. The pre-mRNA undergoes splicing, including removal of the exons (exon skipping), followed by reverse splicing that leads to the formation of the circRNA.^250^ In contrast, intron-pairing–driven and RBP-driven circularization events are both direct back-splicing mechanisms, where two flanking introns are brought into close proximity by repeated, short, complementary DNA sequences (ALU elements), other complementary sequences without repetition, or RBPs respectively.^251^

When first identified more than 30 years ago, circRNAs were thought to be non-functional. However, today, they have been acknowledged to have several important functions, such as acting as miRNA sponges, regulating host gene expression, interacting with proteins, and protein transportation.^251^ They can also serve as diagnostic biomarkers and therapeutics because of their higher nuclease stability and longer half-lives in cells.^252^ In their function as miRNA sponges, circRNA act as ceRNAs, modulating miRNA function and regulating transcriptional gene expression.^252^ Some circRNA can also directly regulate the transcription of their parent gene by interacting with the RNA polymerase II complex.^253^ Finally, circRNAs function as protein sponges by providing the binding sites for proteins like RBPs and inhibiting the biological activity of proteins.^254^ Like lncRNAs, circRNAs can also be translated into functional peptides, although this is relatively rare and not fully understood.^252^

Overall, circRNAs are widely studied in the fields of cancer; cardiovascular, neurological, immune-related, metabolic, and infectious diseases; and aging.^254^ However, the association between circRNAs and alcohol use and misuse is an emerging area of research that has only gained attention recently. Most of the studies to date were conducted to identify the circRNAs and their functional outcomes in AUD, including differential expression of circRNAs in prenatal alcohol exposure,^243^,^255^,^256^ ALD,^257^,^258^ and alcohol dependence^259^ (Appendix 5). circRNAs have also been reported to influence neurotransmitter and signal-transduction pathways after chronic intermittent alcohol exposure,^260^ and to regulate cell survival and apoptosis.^261^ Various studies have investigated the differential expression of circRNAs in animal models, such as mice and rats, and in humans. A study on human serum and exosomes identified hsa_circ_0004771 as a potential biomarker for alcohol dependence (as defined in the *Diagnostic and Statistical Manual of Mental Disorders, Fourth Edition*).^259^ A number of circRNAs have been found to be regulated by prenatal alcohol exposure. Thus, downregulation of circHomer1 occurred in the brain of prenatally exposed adult male mice,^243^ and sex-specific upregulation of circSatb2 and downregulation of circPtchd2 was found in the brain of prenatally exposed male mouse pups.^256^ Moreover, upregulation of circVopp1 in both blood leukocytes and spinal cord tissues but downregulation of spinal immune-regulatory circRNAs, circItch and circRps6ka3, was found in prenatally alcohol exposed adult rats.^255^ The interactions of circRNA with miRNAs such as circRNA-406742 and miR-1200^262^ or circRNA-1639 and miR-122,^257^ indicates a potential role of circRNAs as ceRNAs by acting as miRNA sponges, thereby modulating miRNA activity and thus influencing translation of gene networks associated with AUD.

These scoping analyses of studies assessing circRNAs in AUD highlight several key gaps in the current understanding of the circRNAs’ roles. Thus, little is known about the link between alcohol and the biology of circRNAs. Another important area that deserves greater focus is diagnosis—the extent to which circRNAs predict AUD onset or progression and are biomarkers for treatment effectiveness. Research also is needed to understand how alcohol influences stability, regulation, localization, degradation, and modification of circRNAs. Although general models for circRNA biogenesis exist, the specific impact of alcohol on these processes remains largely unexplored. Although studies have shown associations of circRNA with AUD along with their functional outcomes, more research of individual circRNAs, but more importantly, coordinately regulated groups of circRNAs is necessary to understand their contributions to molecular and cellular processes affected by alcohol. Further, the existing research on circRNA interaction with other ncRNAs, such as miRNAs and lncRNAs, is quite limited, as is data on their interactions with proteins. Each of these areas warrants further investigation.

## Discussion

### Summary of Evidence

This scoping review identified 338 primary research studies addressing the contribution of ncRNAs to alcohol use and misuse and related pathologies across the lifespan, spanning tissues and organs from brain to heart, liver, gastrointestinal system, musculoskeletal system, and placenta. In contrast, a PubMed search using the terms “(Protein) AND (Alcohol Use Disorder)” recovered more than 14,900 results, suggesting that the dominant focus to date has been on protein-based mechanisms. The human genome is estimated to contain 19,411 protein-coding genes; in contrast, ncRNAs are encoded from approximately 43,675 gene loci,^6^ and protein-coding gene loci also encode ncRNAs such as circRNAs, miRNAs, and antisense RNAs. Thus, it is evident that research on ncRNA biology in AUD is in its infancy. Moreover, most research has focused on very few classes of ncRNAs. For instance, research on miRNAs accounted for approximately 86% of all published ncRNA research in AUD in this scoping review. Additionally, a majority of studies at this stage have focused on enumerating ncRNAs that are differentially regulated, and a few ncRNAs, primarily miRNAs such as members of the Let7 family, miR-9, and miR-155, have appeared repeatedly in analyses. This means that the majority of ncRNAs remain to be studied, and entire ncRNA classes such as tRNAs and tRNA fragments, snRNAs, and snoRNAs, remain largely unstudied. Significant gaps in the literature remain, particularly regarding the mechanisms by which ethanol regulates ncRNA expression at both the genomic and post-transcriptional levels. Also, increasing evidence suggests that lncRNAs often express short open reading frames and have the capacity to generate micropeptides under defined biological conditions, an outcome that is completely unstudied. Similarly, it remains unknown what other mechanisms ncRNAs mediate. It is increasingly clear that the functions of individual ncRNAs may be determined by their location within cellular compartments. When localized to the nucleus, an ncRNA may control transcription or splicing, whereas when localized to the cytoplasm, the same ncRNA may control mRNA translation. Another question is whether there are unique, nonparadigmatic mechanisms, for instance, secretion of ncRNAs into biofluids that lend themselves to not only better diagnosis, but also to intervention.

The contribution of key biological variables, such as genetic sex; race and ethnicity; and co-occurring AUD risk factors such as psychological states, environmental stress, and polysubstance use, all remain to be explored. It should be noted that about 19% of the assessed studies did not report the subjects’ genetic sex, and about 41% of studies only used males.

A majority of studies used methodologies such as PCR and microarrays that, while highly quantitative, are insensitive to transcript variations in individual ncRNAs. Only about 5% of studies used RNA sequencing-based approaches, which might yield information about individual transcript variation but were not analyzed for this purpose. Identifying sequence variations within individual ncRNAs is important because they are known to result in functional variation.

Overall, the extent to which ethanol regulates ncRNA expression and function remains incompletely understood. While some studies have documented that specific ncRNAs are modulated by ethanol exposure, the broader regulatory mechanisms and functional consequences of these changes are largely unknown. This is true particularly in the context of cellular metabolism and transcription, which require further investigation. Given the recency of this field—60% of the papers reviewed here were published from 2018 to 2023—it is clear that the immediate next steps are within the domain of the biomedical research community. However, the emerging data on ncRNAs that are secreted into biofluids suggest that their use in diagnostics and particularly, predictive modeling of disease progression is likely to have a significant impact on treatments at all stages of AUD pathology. Further in time lies the promise of ncRNAs as therapeutics. As the field of ncRNA research matures, researchers will increasingly understand the principles of ncRNA action that result from both their primary sequence and their capacity to fold and interact with other cellular components, enabling them to design novel RNA molecules. Moreover, as the roles of chaperones such as lipoproteins and microvesicles are better understood, investigators will be positioned to merge RNA design and delivery strategies to intervene in disease processes.

### Limitations of the Review

The initial search strategy, including selection of search terms, may have resulted in the elimination of informative studies on alcohol effects on ncRNAs. Other equally informative papers may have also been inadvertently omitted. A unique limitation to this review is that because the field of ncRNA biology is new, terminology and gene annotations have evolved over time. For instance, early studies on miRNAs did not discriminate between the 5′ and 3′ strands of pre-miRNAs, which can generate unique miRNAs, and passenger strand miRNAs were annotated with an asterisk. While the 5′ strand is often the retained guide strand, and the 3′ strand is often the eliminated passenger strand, this is not always the case. Moreover, gene annotations have evolved over time, and with that, so have classifications of RNA transcripts as ncRNAs. The continued evolution of this field lends some degree of ambiguity to the published literature, precision of search terms, and data interpretation. Finally, the search may be subject to other publication biases, as it only includes literature published in English, potentially overlooking significant studies in other languages; additionally, biases may arise from selective publication of positive findings or limited access to unpublished studies.

## Conclusions

This scoping review identified 338 research publications that linked ncRNAs to alcohol use. While some studies established associations, others established causal links. Analysis using citation impact tools such as iCite suggests that these studies have a significant and growing impact on research into alcohol use, and the translation potential of these studies is strong. Moreover, these studies link ncRNAs to every stage of alcohol use and misuse, from the induction of acute tolerance to alcohol-associated pathologies, cancer, and FASD. Nevertheless, substantial gaps remain in the knowledge base. ncRNA-coding genes outnumber protein-coding genes, and protein-coding genes can also transcribe ncRNAs. Moreover, ncRNAs can be edited, processed, transferred between sub-cellular compartments, or even secreted into extracellular spaces, where they may act as endocrine molecules. The majority of ncRNAs and their associated mechanisms remain unexplored. Finally, ncRNAs can exhibit dramatic changes with evolution and speciation, and important ncRNAs exhibit sex-specific or sex-biased patterns of expression and function. Consequently, in this field, significant attention needs to be paid to the impact of key biological variables, genetics, and species differences when investigating the linkage between ncRNAs and alcohol use and its consequences.

**KEY TAKEAWAYS**
- Noncoding RNAs (ncRNAs) dominate the genomes of mammals, including humans, and epigenetically transduce the effects of the environment into all cells and tissues.
- Understanding of the role of ncRNAs in the pathogenesis of alcohol use disorder (AUD) is in its infancy.
- Most of the research has focused on very few classes of ncRNAs, mainly on microRNAs.
- Research has mainly focused on documenting ncRNAs that are altered in various AUD states. Very little research has examined ncRNA mechanisms that may mediate AUD pathology and ncRNA roles in interventions.
- Key biological variables such as genetic sex and age are poorly represented among the published studies.
- The field of ncRNA biology is likely to support paradigm shifts in the understanding of AUD pathology and will support novel interventional mechanisms.

## Figures and Tables

**Table 1:** Outline of the Search Strategies for the Medline and Embase Databases

Set	Ovid Medline — Search Statement	Embase — Search Statement
1.	exp microRNAs/	exp microRNA/
2.	exp RNA, Long Noncoding/	long untranslated RNA/
3.	Ethanol/	alcohol/
4.	exp Alcohol-Related Disorders/	exp alcoholism/
5.	exp Alcohol Drinking/	exp drinking behavior/
6.	(microRNA* or miRNA* or circularRNA* or circRNA* or lncRNA* or ncRNA * or (long and non-coding and RNA*) or (long and noncoding and RNA*) or (non-coding and RNA*) or (noncoding and RNA*)).ab,ti.	(microRNA* or miRNA* or circularRNA* or circRNA* or lncRNA* or ncRNA * or (long and non-coding and RNA*) or (long and noncoding and RNA*) or (non-coding and RNA*) or (noncoding and RNA*)).ab,ti.
7.	(alcohol* or ethanol*).ab,ti.	(alcohol* or ethanol*).ab,ti.
8.	exp Humans/	exp human/
9.	exp Animals/	exp animal/
10.	(human* or animal* or rat* or mice* or mouse*).ab,ti.	(human* or animal* or rat* or mice* or mouse*).ab,ti.
11.	1 or 2 or 6	1 or 2 or 6
12.	3 or 4 or 5 or 7	3 or 4 or 5 or 7
13.	8 or 9 or 10	8 or 9 or 10
14.	11 and 12 and 13	11 and 12 and 13
15.	limit 14 to English language	limit 14 to English language
16.		limit 15 to (article or article in press or “preprint (unpublished, non-peer reviewed)”)
**Result**	**1,397 results, 5 duplicates in Covidence**	**1,440 results, 960 duplicates in Covidence**

**Table 2:** Outline of the Search Strategy for the Academic Search Ultimate Database

#	Query	Limiters/Expanders	Last Run Via	Results	Action
S1	DE “NON-coding RNA” OR DE “CIRCULAR RNA” OR DE “MICRORNA”	Expanders: Apply equivalent subjects	Interface: EBSCOhost Research Databases	68,733	
		Search modes: Boolean/Phrase	Search Screen: Advanced Search		
			Database: Academic Search Ultimate		
S2	DE “ALCOHOLISM” OR DE “ALCOHOL” OR DE “ALCOHOL drinking” OR DE “ALCOHOL-induced disorders”	Expanders: Apply equivalent subjects	Interface: EBSCOhost Research Databases	99,319	EditS2
		Search modes: Boolean/Phrase	Search Screen: Advanced Search		
			Database: Academic Search Ultimate		
S3	TI (microRNA* or miRNA* or circularRNA* or circRNA* or lncRNA* or ncRNA * or (long and non-coding and RNA*) or (long and noncoding and RNA*) or (non-coding and RNA*) or (noncoding and RNA*))	Expanders: Apply equivalent subjects	Interface: EBSCOhost Research Databases	64,329	EditS3
		Search modes: Boolean/Phrase	Search Screen: Advanced Search		
			Database: Academic Search Ultimate		
S4	AB (microRNA* or miRNA* or circularRNA* or circRNA* or lncRNA* or ncRNA * or (long and non-coding and RNA*) or (long and noncoding and RNA*) or (non-coding and RNA*) or (noncoding and RNA*))	Expanders: Apply equivalent subjects	Interface: EBSCOhost Research Databases	105,766	EditS4
		Search modes: Boolean/Phrase	Search Screen: Advanced Search		
			Database: Academic Search Ultimate		
S5	TI (alcohol* or ethanol*)	Expanders: Apply equivalent subjects	Interface: EBSCOhost Research Databases	154,013	EditS5
		Search modes: Boolean/Phrase	Search Screen: Advanced Search		
			Database: Academic Search Ultimate		
S6	AB (alcohol* or ethanol*)	Expanders: Apply equivalent subjects	Interface: EBSCOhost Research Databases	419,970	EditS6
		Search modes: Boolean/Phrase	Search Screen: Advanced Search		
			Database: Academic Search Ultimate		
S7	TI (human* or animal* or rat* or mice* or mouse*)	Expanders: Apply equivalent subjects	Interface: EBSCOhost Research Databases	1,520,845	EditS7
		Search modes: Boolean/Phrase	Search Screen: Advanced Search		
			Database: Academic Search Ultimate		
S8	AB (human* or animal* or rat* or mice* or mouse*)	Expanders: Apply equivalent subjects	Interface: EBSCOhost Research Databases	7,577,310	EditS8
		Search modes: Boolean/Phrase	Search Screen: Advanced Search		
			Database: Academic Search Ultimate		
S9	S1 OR S3 OR S4	Expanders: Apply equivalent subjects	Interface: EBSCOhost Research Databases	123,722	EditS9
		Search modes: Boolean/Phrase	Search Screen: Advanced Search		
			Database: Academic Search Ultimate		
S10	S2 OR S5 OR S6	Expanders: Apply equivalent subjects	Interface: EBSCOhost Research Databases	448,388	EditS10
		Search modes: Boolean/Phrase	Search Screen: Advanced Search		
			Database: Academic Search Ultimate		
S11	S7 OR S8	Expanders: Apply equivalent subjects	Interface: EBSCOhost Research Databases	7,880,218	EditS11
		Search modes: Boolean/Phrase	Search Screen: Advanced Search		
			Database: Academic Search Ultimate		
S12	S9 AND S10 AND S11	Expanders: Apply equivalent subjects	Interface: EBSCOhost Research Databases	529	EditS12
		Search modes: Boolean/Phrase	Search Screen: Advanced Search		
			Database: Academic Search Ultimate		
S13	S9 AND S10 AND S11	Expanders: Apply equivalent subjects	Interface: EBSCOhost Research Databases	521	EditS13
		Narrow by Language: English	Search Screen: Advanced Search		
		Search modes: Boolean/Phrase	Database: Academic Search Ultimate		

**Table 3:** Characteristics of the 338 Studies That Explored the Association Between Noncoding RNAs and Alcohol Use

Characteristics	Number of Studies	Percentage of Studies (*n*=338)
**Publication year**		
2007–2012	26	7.7
2013–2017	109	32.2
2018–2023	203	60.1
**Types of ncRNA**		
microRNAs	289	85.5
Long noncoding RNAs	37	10.9
Circular RNAs	10	3.0
Small ribosomal RNAs	1	0.3
Enhancer RNAs	1	0.3
**Sex**		
Male	141	41.7
Female	27	8.0
Male and female	105	31.1
Not reported	65	19.2
**Species**		
Mouse	84	33.9
Human	76	30.6
Rat	54	21.8
Cells (human, murine, rat, zebrafish)	18	7.3
Zebrafish	5	2.0
Rhesus macaque	9	3.6
Drosophila	1	0.4
Sheep	1	0.4
Multiple species (e.g., mouse, rat, cells, and/or human)	90	36.3
**Sample type**		
Brain	79	27.0
Liver, liver cells	58	19.8
Blood, plasma, serum	45	15.4
Cells	42	14.3
Blood, serum, plasma, liver, cells, or brain	41	14.0
Carcinoma and cell line	17	5.8
Extracellular vesicles	8	2.7
Intestinal epithelial cells	7	2.4
Heart, cardiomyocytes	7	2.4
Lung	6	2.0
Embryo	5	1.7
Others	23	7.8
**RNA extraction method**		
Trizol	110	32.5
miRNeasy kit	59	17.5
mirVana isolation kit	44	13.0
RNeasy kit	19	5.6
Trizol / miRNeasy kit / mirVana isolation kit / RNeasy kit	13	3.8
Direct-Zol™ RNA Kit	7	2.1
Exosome isolation kit	3	0.9
miRCURY kit	4	1.2
Others	50	14.8
Not reported	29	8.6
**Method of noncoding RNA analysis**		
Quantitative reverse transcription polymerase chain rection (qRT-PCR)	174	51.5
miRCURY LNA Microarray / qRT-PCR / RNA seq / Affymetrix Arrays / CircRNA Array / LNA Array / Taqman miRNA	43	12.7
Reverse transcription polymerase chain reaction (RT-PCR)	41	12.1
miRNA / circular / lncRNA Arrays	22	6.5
RNA sequencing	17	5.0
TaqMan miRNA assays	14	4.1
Affymetrix arrays	9	2.7
miRCURY LNA Assays	4	1.2
Others	9	2.7
Not reported	5	1.5

**Appendix 1:** Summary of Studies Exploring the Association Between microRNAs (miRNAs) and Alcohol Use in Cancer

miRNAs	Regulation	Cancer Type	Key Finding*	First Author, Year
miR-6819, miR-6877	↑	Alcohol-associated HCC	miR-6819 and miR-6877 regulate ACTG1 and TLR3 gene expression	Gao, 2019^263^
miR-483	↓	Alcohol-associated fatty liver disease and HCC	miR-483 modulates progression of fatty liver disease and inhibits HCC cell proliferation	Niture, 2023^264^
miR-96, miR-126	↑, ↓	HBV tumors, alcohol-related HCC	miR-96 upregulated in HBV tumors; miR-126 downregulated in alcohol-related HCC	Ladeiro, 2008^265^
miR-22-3p	↑	HCC	miR-22-3p promotes HCC stemness and metastasis; TET2 is a downstream target	Chen, 2021^266^
miR-1973, miR-3667-5p, miR-3196	↑	Liver cirrhosis, HCC	miR-1973, miR-3667-5p, and miR-3196 show significant fold change between cirrhosis and HCC samples	Felgendreff, 2020^267^
miR-223, miR-944	↑	HBV-associated HCC	miR-223 correlates with residual tumor presence and poorer survival outcome; miR-944 correlates with mutated proto-oncogenes in HCC tumors; miR-944 inhibitor upregulates tumor suppressor genes	Zheng, 2017^268^
miR-146a-5p	↓	Prostate cancer	miR-146a-5p reduced in exosomes from ethanol-treated cancer-associated fibroblasts	Zhang, 2020^269^
miR-375	↑	HNSCC	Alcohol consumption increased miR-375 levels in HNSCC tissues	Avissar, 2009^270^
miR-30a-5p, miR-934, miR-3164, miR-3178, miR-133a, miR-3138	↑	HNSCC	Alcohol increased miRNAs in oral epithelial cells and HNSCC patients	Saad, 2015^271^
miR-143, miR-203, miR-205	↓	Esophageal squamous cell carcinoma	Lower expression of miR-143, miR-203, and miR-205 in drinkers with esophageal squamous cell carcinoma compared to nondrinkers	Stánitz, 2015^272^
miR-183	↑	Tongue carcinoma	miR-183 associated with clinical stage, tumor size, and high alcohol intake	Supic, 2018^273^
miR-34b/c	↓	OSCC	miR-34b/c promoter methylation associated with nodal status and shorter overall survival	Supic, 2022^43^
miR-34a, miR-99a, miR-143, miR-380-5p	↓	OSCC	Lower expression of miR-34a, miR-99a, miR-143, and miR-380-5p in OSCC samples	Manikandan, 2015^274^
let-7b	↑	Rectal cancer	Significantly increased let-7b miRNA in patients with alcohol-associated liver disease	Mullany, 2017^275^
miR-143	↓	Bladder cancer	Lower miR-143 in bladder cancer patients correlated with tobacco use and chronic alcohol consumption	Boubaker, 2020^276^
miR-126-3p	↓	Cholangiocarcinoma	Marked underexpression associated with smoking, alcoholism, hypertension, and diabetes	Poleto Spinola, 2021^277^

* For definitions of alcohol drinking or exposure levels used, see the original studies cited.

*Note*: HBV, Hepatitis B virus; HCC, hepatocellular carcinoma; HSNCC; head and neck squamous cell carcinoma; OSCC, oral squamous cell carcinoma.

**Appendix 2:** Summary of Studies Exploring the Association Between microRNAs (miRNAs) and Alcohol Use in Alcohol-Associated Systemic Health Problems

Species	Sex	Sample	RNA Extraction	Methods of miRNA Analysis	Key miRNA Findings*	First Author, Year
**A. Musculoskeletal health**						
Human	M/F	Blood, serum	Trizol	miRCURY LNA microarray kit, qRT-PCR	miR-1, miR-127-3p, miR-483-5p, miR-483-3p, miR-628-3p, and miR-885-5p had high diagnostic value for alcohol-induced osteonecrosis of the femoral head.	Hong, 2019^48^
Zebrafish (AB strain)	M	Dorsal epaxial musculature	miRNeasy mini kit	Affymetrix zebrafish miRNA_4.0 array	miR-140-3p exhibited a drastic decrease while miR-146a was elevated; functional enrichment analysis linked miR-140 targets (DNER, JAG1, Hey1) and miR-146a target (Numb) strongly to Notch signaling and muscle differentiation.	Khayrullin, 2016^47^
C57BL/6 mice, human cells	M	BMSCs, HUVECs	Trizol Up kit, Illumina HiSeq 2500	RT-PCR	miR-19a-3p was reduced in HUVECs and BMSCs following ethanol exposure; luciferase reporter assay showed that FOXF2 was the direct binding target of miR-19a-3p; in vivo, miR-19a-3p Agomir stimulated callus transformation and improved the alcohol-impaired fracture healing.	Zhu, 2022^278^
Rhesus macaques	M	Adipose tissue, skeletal muscles, primary cells, EVs	miRNeasy mini kit	qRT-PCR	Chronic binge alcohol use significantly reduced miR-let-7a expression in adipocyte-derived EVs and miR-16 in myotube-derived EVs; higher expression of miR-133a and miR133b was found in myotube-derived EVs.	Bourgeois, 2023^279^
C57BL/6 mice, human cells	M	Femurs, HEK293T, human BMSCs, HUVECs	EZBioscience	qPCR	Alcohol exposure decreased miR-4286 levels in BMSCs and HUVECs and impaired osteogenic differentiation (COL I, OCN, and OPN); treatment with miR-4286 reversed these inhibitory effects.	Yu, 2021^44^
C57BL/6 mice, human cells	M	Femur, HUVECs, BMSCs	Trizol-up	RT-PCR, miRNA mimic	miRNA-136-3p was reduced in alcohol-induced osteopenia, which suppressed osteogenic differentiation in BMSCs and vascularization in HUVECs; miR-136-3p agomir ameliorated those effects; miR-136-3p also targeted phosphatase and PTEN in cell groups.	Chen, 2020^45^
Human	M/F	Bone marrow	RNeasy kits	RT-PCR	miR-31 was highly expressed in osteonecrosis tissues and negatively regulated the SATB2/RUNX2 pathway; TNF-alpha inhibitor induced miR-31 decrease and enhanced osteogenic differentiation.	Yu, 2019^46^
Sprague Dawley rats	M	Right hind limb	Trizol	Microarray	20 microRNAs were increased, and six microRNAs were decreased, with alcohol exposure.	Sampson, 2011^280^
**B. Cardiovascular disease**						
Human cells		AC16 cardiomyocytes	Trizol	qRT-PCR	Ethanol exposure increased miR-186-5p in AC16 cardiomyocytes and decreased its target gene XIAP, promoting cardiomyocyte apoptosis.	Liu, 2019^281^
Rat (unspecified)		Heart, primary cardiomyocytes	Trizol	qRT-PCR, Taqman Advanced miRNA Assays	Ethanol induced apoptosis in primary cardiomyocytes by upregulating miR-378a5p, which reduced ALDH2.	Wang, 2017^282^
Friend Virus-B mice	M	Heart	miRNeasy kit	microRT reverse transcription kit	Chronic alcohol consumption increased cardiac miR-214, reducing Bcl-2 levels; sildenafil suppressed miR-214, attenuating its pro-apoptotic effects.	Samidurai, 2020^283^
Rat cells		H9C2 cardiomyocytes	RNAiso Plus	qRT-PCR, miRNA mimic	Ethanol increased miR-155-5p in H9C2 cells, improving insulin sensitivity and reducing ethanol-induced insulin resistance; miR-155 regulated the mTOR pathway and its decrease increased p-MTOR levels, attenuating myocardial insulin resistance.	Li, 2020^50^
Human	M/F	Blood	Trizol	qPCR	miR-133b was decreased in patients with alcohol-associated cardiomyopathy compared to controls; miR-138 in patients correlated with increased ejection fraction and left atrium diameter.	de Araújo Melo, 2020^284^
Kunming mice	M	Heart tissue	Trizol	qRT-PCR	Ethanol increased miR-21 and miR-221 in myocardial tissues; hydrogen sulfide treatment significantly decreased these miRNAs and could reverse myocardial fibrosis, collagen deposition, and autophagy caused by long-term alcohol exposure.	Liang, 2017^285^
Human	M	Plasma	Trizol	RT-PCR	Alcohol exposure dysregulated 21 miRNAs in cardiac tissues, with hsa-miR-138 being significantly upregulated.	Jing, 2015^178^
C57BL/6 mice	M	Heart tissue	mirVana miRNA Isolation Kit	qRT-PCR	Ethanol dysregulated 19 miRNAs; miR-151-5p, miR-322-5p, and miR-223-5p targeted oxidative stress-related genes (Nfe2l2, Atf3/4, Klf5), exacerbating myocardial injury; ethanol consumption also reduced ejection fraction and disrupted lipid metabolism.	Xue, 2023^286^
Friend Virus-B mice/Rat cells	M/F	Heart, ventricle, H9C2 cardiomyocyte	mirVana qRT-PCR miRNA Isolation and Detection Kit	qRT-PCR	miR-30a regulated autophagy via Becn1; chronic alcohol intake with the ADH transgene decreased miR-30a and Becn1, contributing to contractile dysfunction.	Guo, 2012^287^
Wistar rats	M	Blood, brain tissue	Trizol	qRT-PCR	miR-219 expression was reduced in brain tissue and blood in alcohol and alcohol+ischemia groups; NMDA expression in the brain was increased in these groups, indicating an inverse correlation between NMDA modulation and miR-219.	Silva, 2017^288^
Human	M	Blood, urine	miRCURY™ RNA Isolation Kit-Biofluids	qRT-PCR	Alcohol intake increased miR-155-5p, miR-328-3p, and miR-92a-3p; miR-145-5p, miR-17-5p, miR-20-5pa, miR-26b-5p, and miR-223-3p levels were increased in macrophage after alcohol consumption.	Daimiel, 2020^53^
Wistar rats	M	Blood	RNAiso Plus	qRT-PCR, AAV-miR-155-5p	Chronic alcohol intake upregulated miR-155-5p, which ameliorated myocardial insulin resistance by downregulating the mTOR signaling pathway.	Li, 2021^51^
Wistar rats	M	Serum, cerebellum		RT-PCR	miR-126 and miR-155 were increased in alcohol and alcohol+ischemia groups; caspase-9 was increased in cerebellum Purkinje cells in the alcohol+ischemia group.	Silva, 2019^289^
Kunming mice	M	Heart tissue	Trizol/RNeasy mini kit	miRCURYTM Hy3/Hy5 power labeling kit	Myocardial changes were found in chronic alcohol-fed mice, such as fatty acid accumulation, cardiomyocyte vacuolization, myocardial myofibril loss and disarray, sarcoplasmic reticulum edema, and swollen disrupted mitochondria.	Yang, 2018^290^
**C. Gastric epithelial injury**						
Human cells	M/F	Colon, Caco-2 intestinal epithelial cells	mirVana RNA isolation kits (Ambion)/RNEasy Kit	Stem-loop RT-PCR (for cells/tissue), qRT-PCR for ZO-1 protein)	Ethanol significantly increased miR-212 in Caco-2 cells, which directly reduced ZO-1 protein levels, causing disruption to intestinal barrier integrity.	Tang, 2008^58^
C57BL/6J mice, human cells		Liver, colon, Caco-2 intestinal epithelial cells	mirVana RNA isolation kits	Stem-loop RT-PCR	Inhibition of miR-212 overexpression in alcohol treated Caco-2 cell monolayers and in alcohol-fed mice by miRNA knockout prevented alcohol-induced intestinal hyperpermeability.	Tang, 2015^59^
C57BL/6 mice	M	Intestinal tissues, liver	Trizol	qRT-PCR, miRNA inhibitor, hematoxylin & eosin staining	Alcohol increased miR-141 in intestinal tissues; miR-141 reduced inflammatory markers TNF-alpha/IL-6 as well as mRNA and protein levels of Tlr4, Cd14, and NF-kappa-B p65; using these mechanisms, miR-141 ameliorated AH-induced pathological changes.	Qian, 2019^291^
Balb/c mice	M	Gastric tissue	Trizol	qRT-PCR	Ethanol increased miR-9-5p, which led to gastric ulcer; treatment with *Paecilomyces sinensis* significantly reduced miR-9-5p expression; this treatment also promoted anti-inflammatory effects and improved integrity of gastric mucosa.	Zhou, 2021^60^
Human cells		Human gastric epithelial cell line (GES-1)	Trizol	qRT-PCR	EtOH reduced miR-21, but pre-treatment with ghrelin caused increases in miR-21, which protected against apoptosis in GES-1 cells; antagomir-21 reversed protective effects of ghrelin, which suggests role of miR-21 in anti-apoptotic effects.	Jiang, 2015^292^
Human cells		Primary human gastric epithelial cells, and GES-1 cells	TOYOBO ReverTra Ace-a RT-PCR kit	qRT-PCR	GES-1 cells expressing antagomir-451 had protection from the effects of ethanol; antagomir-451 inhibited ethanol-induced apoptosis and oxidative stress in GECs due to AMPK activation for apoptosis that was caused by the depletion of miR-451 by its antagomir.	Zhu, 2018^62^
Sprague-Dawley rats, human cells	M	Gastric mucosal tissue, GES-1 cells	Trizol	qRT-PCR, miRNA mimic	Ethanol increased miR-145 and reduced miR-17, miR-19a, miR-21, miR-181a, and miR-200c in gastric tissues; miR mimics confirmed that miR-19a and miR-21 had an anti-apoptotic effect while miR-145 had a pro-apoptotic effect; ethanol activated JNK pathway and a JNK inhibitor attenuated ethanol-induced GES-1 apoptosis.	Luo, 2013^64^
C57BL/6 mice	F	Serum, small intestine	RNeasy kit	qRT-PCR	miR-155 was increased in chronic alcohol-fed mice; miR-155 deficiency prevented NF-kappa-B activation, TNF-alpha mRNA, and protein expression in the small bowel increased following chronic alcohol feeding, which prevented alcohol-induced increase of serum endotoxin and bowel inflammation.	Lippai, 2014^293^
WT and TLR4-KO mice (C57BL/6)	M	Feces, PFC, colon	Trizol, QIAamp Fast DNA Stool Mini Kit	qRT-PCR	Ethanol increased miR-155-5p, miR-146a-5p, and inflammatory genes (IL-1beta, iNOS, TNF-alpha); ethanol altered structural and permeability genes (INTL1, CDH1, CFTR) in the colon of WT mice; TLR4 path deficiency mitigated the pro-inflammatory response and partially preserved gut microbiota.	Cuesta, 2021^61^
C57BL/6N mice, human cells	M	Ileum, liver, Caco-2 cells	Trizol	qRT-PCR	Alcohol increased miR-122a in the ileum, causing reduction of occludin protein; *Lactobacillus rhamnosus* inhibited miR-122a, which protected from ALD by reducing inflammation and maintaining intestinal barrier integrity.	Zhao, 2015^294^
**D. Pancreatic/metabolic disease**						
Human	M	Serum	miRNAEasy kit	miRNA 4.0 Arrays	72 miRNAs were upregulated and 78 were downregulated in patients with alcohol-associated chronic pancreatitis compared to people with AUD and no pancreatitis (miR27a-3p had the highest upregulation/fold change); this dysregulation caused significant changes in both pro- and anti-inflammatory pathways.	Chhatriya, 2020^66^
C57BL/6 mice	M	Pancreatic stellate cells	microRNeasy Plus Mini Kit	qRT-PCR	Upregulation of CCN2 expression levels by activated pancreatic stellate cells drove miR-21 induction, which acted via positive feedback loop to potentiate CCN2 production, thereby amplifying collagen production in the cells.	Charrier, 2014^67^
Human	M/F	Serum	Trizol	qRT-PCR	Increased miR-330 was observed in patients with AUD and type 2 diabetes mellitus.	Ali Beg, 2020^71^
C57BL/6 mice	M	Liver, serum	NucleoSpin miRNA mini kits	RT-PCR	Increasing concentrations of ethanol in high-fat diet groups caused upregulation of miR-29; this upregulation was associated with a lower liver/body weight ratio, inflammation, fat deposition, and fibrosis; upregulation caused less severe liver injury.	Liang, 2023^295^
Sprague-Dawley rats	M/F	Liver, abdominal adipose tissue, plasma	miRNeasy Mini Kit	TaqMan Advanced miRNA assays	Periconceptual alcohol exposure caused increased miR-29b (males only) and reduced miR-26a in abdominal adipose tissue, which contributed to changes in metabolic profiles; males showed increased susceptibility to these changes.	Gårdebjer, 2018^296^
Sprague-Dawley rats, murine cells	M	HT22 cells, rat whole brain and PFC	RNeasy kit	SDS software, Agilent 2100 bioanalyzer	Chronic ethanol consumption led to the increase of miR-let-7, leading to mitochondrial deficit.	Jung, 2015^297^
**E. Alcohol-associated liver disease**						
Sprague-Dawley rats		Liver	mirVana™ miRNA Isolation kit	miRCURY LNA Microarray kit, stem-loop qPCR	In ASH, miR-129 showed the largest increase and miR-199a-3p the largest decrease; in AFLD, miR-200c showed the largest increase and miR-93 the largest decrease; Prediction Analysis of Microarrays-selected miRNA profile provided almost 100% diagnostic accuracy of the different stages of ALD when compared with liver biopsy.	Chen, 2014^298^
Human	M/F	Blood, plasma		HTG EdgeSeq miRNA Whole Transcriptome Assay	Alcohol consumption increased miR-193b3p, miR-122-5p, and miR-3937 and decreased miR-4507.	Karabegović, 2023^299^
C57BL/6 mice, murine cells	M	Liver, AML-12 cells	Trizol	qRT-PCR	miR-203 decreased in alcohol-fed mice and ethanol-treated cells; overexpression of miR-203 reduced lipid accumulation by targeting Lipin1, a regulator of hepatic lipid metabolism, thus reducing AFLD progression.	Cheng, 2018^83^
Human, C57BL/6 mice, human cells	M/F	Liver, Huh-7	miRNeasy kit	qRT-PCR	Chronic alcohol use reduced miR-122 in human livers and murine hepatocytes; alcohol decreased miR-122 by increasing GRHL2; ALD induced by direct miR-122 inhibition could be prevented in hepatocyte-specific HIF1A-deficient mice.	Satishchandran, 2018^72^
C57BL/6 mice	F	Plasma, serum	miRNAEasy kit	TaqMan miRNA assay	Serum/plasma miR-122 and miR-155 could serve as biomarkers of liver damage and inflammation, respectively.	Bala, 2012^73^
Human	M/F	Blood	Phenol/chloroform extraction	qRT-PCR	miR-513-3p and miR-571 increased, and miR-652 decreased in the serum of patients with alcohol-associated or hepatitis C-induced liver cirrhosis; miR-571 was associated with alcohol-related cirrhosis progression.	Roderburg, 2012^82^
Sprague–Dawley rats, LX-2 human HSC line	M	Liver, cells	miRNeasy Mini Kit	qRT-PCR, LNA oligonucleotide probe antisense to miR-21	Chronic ethanol consumption altered miRNA profiles including a decrease in miR-21; in vivo inhibition of miR-21 correlated with changes in miR-340-5p and inversely correlated with miR-365, let-7a, miR-1224, and miR-146a after partial hepatectomy.	Parrish, 2021^77^
Human, human cells	M/F	Liver, L02 cells	Trizol	miRNA qRT-PCR detection kit	miR-708 was reduced in human ALD liver tissues compared with healthy controls; expression levels of TNF-alpha and IL-6 were induced in ethanol-induced L02 cells, and miR-708 could reduce the secretion of TNF-alpha and IL-6; protein expression level of ZEB1 in L02 cells was negatively regulated by alteration of miR-708; AKT/mTOR signaling pathway was activated when ZEB1 expression was downregulated.	Hu, 2020^300^
LTG mice		Blood		qRT-PCR	miR-26a promoted autophagy; overexpression of miR-26a in mouse liver alleviated ethanol-induced hepatic steatosis and liver injury.	Han, 2015^84^
Human, C57BL/6 mice	M	Liver		qRT-PCR	miR-148a decreased in patients with AH; marked suppression of FoxO1 in the liver of patients with AH and ALD animal models; FoxO1 transcriptionally controlled miR148A expression by binding to promoter sites.	Heo, 2019^301^
Human	M/F	Plasma, liver		qRT-PCR/TaqMan microRNA assays	miR-21-5p, miR-24-3p, and mir-146a-5p increased according to fibrosis severity in regulating the sphingolipid pathway.	Thiele, 2023^302^
Zebrafish	M/F	Liver, blood	RNeasy Mini Kit	qRT-PCR	miR-122 and miR-155 increased in liver; miR-155 and miR-217 increased in blood; dysregulation of these miRNAs affected cytokine expression.	Pasqualotto, 2021^303^
Long Evans rats, human cells	M	Liver, OUMS29 cells	miRNA isolation kit	QRT-PCR	miR-34a increased in the livers of ethanol-fed rats; miR-34a significantly reduced sirtuin 1 expression, which has been implicated in promoting cell survival and cell growth in hepatocytes; overexpression of miR-34a inhibited the expression of anti-apoptotic protein, Bcl2.	Iwagami, 2018^79^
Human hepatocytes		Cells	Direct-zol™ RNA MiniPrep kit	TaqMan miRNA assays	Alcohol induced the transcription of various Rab proteins and of v-SNARE and t-SNARE proteins in human hepatocytes and in people with ALD; alcohol reduced miR-192 and promoted exosome secretion; miR-192 regulated exosome secretion in human hepatocytes via targeting Rab27a, Rab35, and syntaxins.	Bala, 2022^304^
Human, C57BL6 mice		Blood, liver (hepatic macrophages and hepatocytes), EVs	miRNAEasy kit	Small RNA sequencing, qRT-PCR	let-7f, miR-29a, and miR-340 levels increased in blood EVs from ASH mice, but not in other chronic liver injury models. These levels were also higher in patients with mild ALD compared to people without AUD.	Eguchi, 2017^305^
Human, C57BL/6 mice	M/F, M	Plasma (humans), blood, liver (mice)	Trizol	qRT-PCR	Baicalin relieved ALD and increased miR-205 expression in liver tissues of ALD mice; miR-205 inhibited importin-alpha5 expression, thereby inactivating the NF-kappa-B signaling pathway and attenuating the progression of ALD.	Fang, 2022^85^
Human, C57BL/6 mice	M/F, M	Liver (human), serum, hepatocytes (mice)	mirVana miRNA Isolation Kit	qRT-PCR	Chronic ethanol exposure increased Sct/SR signaling that was able to reduce the expression of miR-125b.	Kyritsi, 2023^306^
Mice		Liver	RNA Isolation Kit	RT-PCR	A bicolor fluorescent nanoprobe was developed to efficiently detect AFLD and ASH by simultaneously imaging miR-155 and osteopontin mRNA in vivo.	Yang, 2017^307^
HNP-1 transgenic mice, C57BL/6N mice	M	Blood/Liver	miRNeasy Mini Kit	miScript miRNA PCR array	After 24-week ethanol intake, miRNA-34a5p increased in liver of transgenic mice compared to WT mice.	Ibusuki, 2017^75^
Human cells		HepG2, Huh7, and 293T cells	Trizol	qRT-PCR, miRNA mimic, in silico bioinformatics	hsa-miR-1301-3p suppressed the expression of ADH6, ALDH5A1, and ALDH8A1 in liver cells and blocked their induction by ethanol; 11 miRNAs that target ADH and ALDH genes were identified by in silico bioinformatics.	Wang, 2020^308^
C57BL/6J mice	M	AML-12 hepatocytes		qPCR	miR-200a increased in mice after chronic and binge ethanol feeding and AML-12 cells stimulated by ethanol; miR-200a induced hepatocyte apoptosis in vivo and in vitro; ZEB2 expression pattern and miR-200a-3p directly targeted and inhibited ZEB2 expression.	Zhao, 2018^309^
C57BL/6 mice, human cells	M	Liver tissue, LO2 cells	RNAiso Plus reagent	qRT-PCR	miR-194 increased in ALD, which was significantly suppressed by yangonin; miR-194 mimic inhibited farnesoid X receptor expression in vitro.	Dong, 2021^310^
Human cells		HSCs	mirVana isolation kit	qRT-PCR	Decreased let-7a and let-7b was associated with HSC activation in ALD mouse livers and in LPS/TGF-beta–treated HSCs compared with controls; silencing of Lin28 in Lin28Bdeficient mice inhibited activation of HSCs and subsequently facilitated tissue recovery in livers of ethanol-fed mice.	McDaniel, 2017^311^
Wistar rats, C57Bl/6J mice	M, F	Kupffer cells, liver tissue	miRNAEasy kit	qRT-PCR	Next-generation sequencing identified three miRNAs whose expression was restored by hyaluronic acid after downregulation due to ethanol feeding; of these miRNAs, miR181b3p was most reduced by ethanol and regulated the expression of importin-alpha5.	Saikia, 2017^312^
C57BL/6J	M	Liver	RNeasy Plus Mini Kit	qRT-PCR	let-7a-5p and miR-21a-5p decreased in the ethanol group; miRNA–mRNA interaction showed inverse relation of Casp-3, Rb1, and Ccng1 with miR-21 and let-7a expression; let-7a inhibition in hepatocytes confirmed apoptosis induction and upregulation of RB1 protein expression.	Pan, 2020^313^
Human hepatocytes		HSCs, HepG2 cells	Trizol	Real-Time PCR	miR-21 increased in mouse livers with ALD after the activation of IL-6/Stat3 signaling in vivo; miR-21 increased in ethanol-treated hepatic cell lines; IL-6 contributed to alcohol-associated liver injury and tissue repair through miR-21 by modulating cell proliferation, apoptosis, and survival.	Francis, 2014^78^
Human		Blood, serum		qRT-PCR	No significant changes occurred in miR-122, miR-125b, or miR-192 in alcohol-related liver injury.	Xu Ping, 2017^314^
C57BL/6 mice, human cells	M	Serum, liver, L02 cells	Trizol	qRT-PCR	miR-378b expression was boosted in ethanol-fed mice and ethanol-triggered L02 cells; at the same time, it was accompanied by insulin signal pathway dysfunction; overexpressed miR-378b reduced PI3K/AKT activation; inhibition of miR-378b improved insulin resistance in vivo and in vitro; miR378b overexpression reduced insulin receptor or p110-alpha expression, whereas the downregulation of miR-378b increased insulin receptor or p110-alpha expression.	Li, 2020^315^
Human, C57BL/6J mice, rat cells	M	Liver, plasma, HSC-T6	RNeasy Mini kit	qRT-PCR	miR-96 and sonic hedgehog (Shh) were upregulated in samples from ethanol fed-mice and people with alcohol-associated hepatitis ; anti-miR-96 and the hedgehog pathway inhibitor MDB5 synergistically intervened in the progression of ALD.	Kumar, 2023^74^
C57BL/6 mice, cells	M	Liver, cryopreserved primary human hepatocytes	Trizol	Real-time qPCR	A let-7 mimic induced TNF-alpha in a human hepatocyte cell line; both TLR7 expression and let-7b expression were increased in patients with AH and positively correlated with expression of IL-8.	Massey, 2018^316^
Human, C57BL/6J mice	M	Liver, blood, cells	Trizol	qRT-PCR	miR-873-5p was identified as a key regulator of liver injury in ALD; inhibition of miR-873-5p reduced nicotinamide N-methyltransferase activity, restored NAD metabolism through the salvage pathway, and enhanced SIRT1 deacetylase activity.	Rodríguez-Agudo, 2023^317^
Human	M	Liver	Trizol	RiboGreen kit, microarray hybridization	26 differentially expressed miRNAs were identified in ALD; increase of hepatic miR-873-5p and decrease of miR-873-5p led to lipid accumulation and hepatocyte death in ALD patients.	Liu, 2013^318^
C57B/6 mice	M	Liver, blood	Trizol and Rnase-free Dnase I or Quick-RNA MiniPrep	RT-PCR	Mice with AH had increased release of hepatocyte-derived EVs (HC-EVs) with a signature set of miRNAs and damage-associated molecular patterns that are involved in proinflammatory and profibrogenic regulation of HSCs and hepatic macrophages; specific AH-HC-EV-derived miRNAs (e.g., miR-27a, miR-181a) targeted selective HSC mRNAs that are involved in HSC quiescence and repressed in AH-HSCs (e.g., Nr1d2).	Eguchi, 2020^319^
Human, C57Bl/6J mice		Cells, liver tissue, normal human hepatocytes	Trizol	RT-PCR	miR-34a was increased in ethanol-exposed mouse liver in vivo and overexpressed in ethanol-treated hepatobiliary cell lines compared with controls; CASP2 and SIRT1 were identified as targets for miR-34a; concomitant miR-34a–dependent activation of metalloproteinases in hepatobiliary cells could facilitate tissue remodeling.	Meng, 2012^80^
C57BL/6J mice, murine cells	M	Liver, RAW264.7 macrophage cells		qRT-PCR	Ethanol increased miR-217 in liver, which caused increase in inflammation through increase in KCs, elevated mRNA levels of proinflammatory cytokines, impaired SIRT1 expression, and activation of NF-kappaB/NFATc4 pathways.	Yin, 2015^320^
C57BL/6 mice, miR-29b-/- mice, murine cells	M/F	Liver, serum, RAW264.7 macrophage cells	RNA simple Total RNA Kit, miRcute, miRNA Isolation Kit	qRT-PCR	Ethanol reduced miR-29b in liver and liver macrophages, which upregulated STAT3 to exacerbate liver inflammation; miR-29b-/mice showed elevated miR-29b levels and reduced STAT3 levels, which could protect against alcohol-associated liver injury.	Zhou, 2022^321^
C57BL/6J mice, human cells	M	Liver, AML12 cells	Trizol	qRT-PCR with QuantiNova™ SYBR Green PCR reagent	miR-214 helped sensitize hepatocytes to ferroptosis by increasing the transcription of key ferroptosis-driver genes, including ACSL4, SLC38A1, and RPKAA2.	Luo, 2023^322^
C57BL/6 mice, human cells	M	Liver, L02cells	Trizol	qRT-PCR	miR-182-5p increased in ALD compared with normal livers, whereas the expression of FOXO1 was decreased by alcohol consumption; dual-luciferase reporter assays demonstrated that miR-182-5p directly targeted the binding site of the FOXO1 3′UTR and inhibited its mRNA and protein expression.	Zuo, 2021^323^
C57BL/6J mice, humans	M	Bone marrow, blood		qRT-PCR	Neutrophilic SIRT1–miR-223 axis was reduced through C/EBP-alpha acetylation in aged mice; similar downregulation was observed in middle-aged/elderly people; the reduction in SIRT1 and miR223 increased inflammatory mediators and ROS, leading to liver injury in mouse model and patients with chronic alcohol drinking.	Ren, 2022^324^
Human, C57Bl/6J mice	M/F, F	Serum, neutrophils	Trizol	qRT-PCR	Chronic ethanol binge drinking increased serum miR-223 and neutrophils in a mouse model; miR-223 directly inhibited IL-6 expression and subsequently inhibited p47phox expression in neutrophils; deletion of the p47phox gene ameliorated ethanol-induced liver injury and ROS production by neutrophils.	Li, 2017^325^
C57BL/6 mice, human cells	M	Serum, liver, L02 cells	PrimeScript® RT kit	qRT-PCR	miR-23a-3p increased in ALD, which was inhibited by isoliquiritigenin (from licorice); the miR-23a-3p inhibitor also increased lipid metabolism in ALD via PGC-1alpha activation.	Wang, 2022^326^
C57BL/6 mice	M	Liver, serum	Trizol	qRT-PCR	Rice bran phenolic extract treatment of ethanol-fed mice reduced miRNA-494-3p, which regulated PGC-1alpha expression directly; rice bran phenolic extract might exert protection against ALD by alleviating mitochondrial dysfunction and the resulting hepatocyte apoptosis via the PGC-1alphaTFAM signal pathway mediated by miRNA-494-3p.	Xiao, 2020^327^
C57BL6/J mice	M/F	Nucleus accumbens	miRVana kit	qRT-PCR with TaqMan Small RNA Assays	Short ethanol exposure increased mature miR-9-5p expression, which was followed by a gradual decrease and subsequent increase of the expression, returning to pre-exposure levels within 24 h; temporal changes of miR-9-3p expression were complementing miR-9-5p changes; extended, continuous presence of alcohol caused a similar pattern.	Mead, 2023^142^
Human, C57BL/6 mice, miR-155 KO mice, murine cells	M/F	Blood, liver, Hepa1–6, RAW264.7 macrophage cells, primary hepatocytes, KCs	miRNeasy isolation kit	qRT-PCR	Ethanol increased miR-155 in mouse and human liver, which impaired autophagy by inhibiting the mTOR pathway; miR-155 targeted lysosome membrane proteins (LAMP1/LAMP2), which reduced lysosomal function; alcohol increased exosome release in a miR-155–dependent manner, while miR-155 KO mice maintained normal levels.	Babuta, 2019^76^
Human cells		Huh7 cells	Trizol	qRT-PCR	miR-29c and ADH6 were both reduced in ethanol-exposed hepatic cells, and exogenous miR-29c increased ADH6 mRNA and protein expressions without affecting the stability of ADH6 mRNA; exogenous miR-29c translocated into the nucleus, bound to the target enhancer DNA sequence, and acted as an enhancer activator that increased ADH gene cluster expression.	Chen, 2022^166^
C57BL/6 mice	F	Liver, blood, HSCs, KCs	Trizol	RT-PCR	Modular CXCR4-inhibiting nanosystem PEIPBA@DA-C (PPC) could be targeted to the liver with the introduction of mannose to help the nanosystem deliver the anti-miR-155 into the KCs to reduce expression of miR-155, thus reducing the activation of KCs as well as the production of proinflammatory cytokines.	Jia, 2023^328^
C57BL/6J mice, murine cells	F	Liver, RAW 264.7 cells	miRNeasy isolation kit	qRT-PCR	Chronic alcohol intake increased miR-155 expression in KCs; miR-155 inhibited negative regulators of the TLR4/LPS pathway in KCs.	Bala, 2017^329^
Human	M/F	Blood, liver	Trizol	RT-PCR	Two single nucleotide polymorphisms, rs361525(G/A) in TNF-alpha and rs1143627(C/T) in IL1-beta were identified as genetic risk factors for ALD susceptibility; restoration of miR-124-3p inhibited TNF-alpha and IL1-beta, along with key genes in TLR4 signaling, apoptosis, and fibrogenesis pathways, effectively reducing hepatocyte injury, fibrosis, and inflammation.	Dasgupta, 2023^330^
Wistar rats	M	Liver	Trizol	RT-PCR	miR-135 and miR-199 decreased in ethanol-fed rats; liver sinusoidal endothelial cells treated with ethanol had reduced miR-135 and miR-199; ethanol-induced ET-1 expression in rat liver sinusoidal endothelial cells was regulated by miR-199, while in human endothelial cells, ET-1 expression was regulated by miR-199 and miR-155, indicating that these microRNAs may function as novel negative regulators of ET-1 transcription.	Yeligar, 2009^331^
C57BL/6 mice	M	Blood, liver	Trizol	RT-PCR	Ethanol increased miR-378b expression while caffeic acid dimethyl ether (CADE) prevented miR-378b increase in vivo; miR378b escalation exacerbated hepatic steatosis and inhibited CaMKK2-AMPK signaling, while miR-378b deficiency alleviated lipid accumulation and activated the CaMKK2 cascade; knockdown of miR378b eliminated the beneficial effect of CADE on lipid metabolism.	Lu, 2022^332^
C57BL/6 mice, murine cells	M	Mouse primary hepatocytes, AML-12 cells	Trizol reagent	qPCR	Melatonin-induced miR-497 decreased alcohol-mediated bile acid synthesis by attenuating the BTG2-YY1 signaling pathway both in vivo and in vitro; this beneficial effect of melatonin was negated by a miR-497 inhibitor.	Kim, 2017^333^
C57BL/6 mice, human HSC cells	F	liver, serum, LX-2 cells	RNAlater	miRCURY LNA miRNA PCR Starter Kit	miR-155 increased in livers affected by alcohol-associated liver disease; miR-155 silencing with Chol-PCX/anti-miR-155 nanoparticles resulted in overall therapeutic benefit.	Zhang, 2022^334^
C57BL/6 mice, murine cells	F	Liver, RAW 264.7 cells	mirVanaTM miRNA isolation kit	qRT-PCR	Chronic alcohol exposure increased TNF-alpha production via miR-155 in liver macrophages; miR-155 overexpression increased TNF-alpha mRNA stability in macrophages even in the absence of alcohol.	Bala, 2011^335^
BALB/c albino mice	M	Liver	Trizol	RT-PCR	Hepatic expression of miR-155 increased in ALD mice but decreased in boswellic acid-treated mice.	Salama, 2023^336^
Human, human cells	M/F	Plasma, primary human monocytes, THP-1 cells	Direct-zol™ RNA MiniPrep isolation kit	qPCR	Alcohol increased EV production in monocytes; alcohol-exposed monocytes communicated with naive monocytes via EVs; miR-27a cargo in monocyte-derived EVs could program naive monocytes to polarize into M2 macrophages.	Saha B, 2016^337^
C57BL/6J mice	M/F	Blood, liver		qPCR	miR-155 KO mice exhibited reduced liver fibrosis markers in both alcohol- and CCl4induced fibrosis; reduction in PPRE and PPAR-alpha binding observed in WT mice was prevented in miR-155 KO mice after alcohol diet; miR-155 inhibition resulted in an increase in PPAR-gamma gene in both naïve and alcohol-treated cells.	Bala, 2016^338^
Human, Wistar rats (chronic ethanol), C57BL6/J mice (short-term ethanol)	M/F	Peripheral blood mononuclear cells, liver, KCs	RNeasy mini kit	qRT-PCR	Ethanol increased 11 miRNAs in KCs, mainly miR-29-1b; hyaluronic acid normalized dysregulation of miR-29, which was correlated with increased Tollip protein expression, mitigating ethanol-induced inflammation.	Saikia, 2017^312^
Human	M/F	Prefrontal and motor cortices, HEK293T cells	Trizol	miScript SYBR Green PCR Kit and the Rotor-Gene Q	miR-203 expression increased in the PFC of people with alcohol-associated cirrhosis, especially females; cotransfection of miR-203 and clones containing the GABRA5 3′-UTR reduced 3′-UTR reporter activity in HEK293T cells.	Janeczek, 2020^339^
Human, BALB/c mice	M/F, M	Plasma, liver	Trizol	qRT-PCR	Plasma miR-122 levels exhibited a disease severity–dependent increase in patients with chronic hepatitis B and in animal models of alcohol-and chemical-induced liver injury; changes in miR-122 appeared earlier and were more specific to liver injury than for other organ damage.	Zhang, 2010^340^
Human, human cells	M/F	Blood, liver, U937 cells	miRNeasy Mini Kit, miRNeasy Serum/Plasma Kit	qRT-PCR	In people with HBV-related ACLF and alcohol-induced ACLF, miR-124a levels increased while GR-alpha expression decreased compared to chronic HBV patients and controls; there were no significant differences in miR-124a levels and GR-alpha expression between HBV-ACLF and alcohol-induced-ACLF patients; miR-124a levels correlated negatively with GR-alpha expression in monocytes and positively with inflammatory factors such as IL-1beta, IL-6, and TNF-alpha.	Wang, 2020^341^
C57BL/6 mice	M	Liver		qRT-PCR	miR-30e decreased in line with the progression of ALD stages; miR-30e over expression in AH caused reduced inflammation, followed by significantly increased ATP and H2O2 levels.	Jin, 2017^86^
Human, C57BL/6 mice	M/F, F	Blood, serum, plasma, exosomes	Direct-zol™ RNA MiniPrep isolation kit	Firefly miRNA multiplex assay (Firefly™ bioworks), qRT-PCR	Consistent with the animal model, total number of EVs (mostly exosomes) increased in people with AH; both miRNA-192 and miRNA-30a increased in the circulation of people with AH.	Momen-Heravi, 2015^87^
Human, Wistar rats	M	Liver	miRNeasy Mini Kit	Small RNA sequencing, qRT-PCR	miRNAs were differentially expressed in primary KCs from ethanol-fed rats; polarization-associated miR-125a-5p was increased.	Kim, 2019^342^
Human, C57BL/6	M/F	Blood, liver	miRNeasy Serum/Plasma kit	miRNA 3.0 Array qRT-PCR	miR-182 expression was elevated in AH, correlating with disease severity and contributing to liver injury.	Blaya, 2016^88^
Human, C57BL/6 mice	M/F	Blood, serum, liver, exosomes	Direct-zo™l RNA MiniPrep isolation kit	TaqMan miRNA Assays, qRT-PCR	In alcohol-fed mice, increased miRNA-122 in liver cells was transferred via exosomes to monocytes, inhibiting heme oxygenase 1, increasing LPS sensitivity, and boosting proinflammatory cytokines; inflammation was prevented by delivering a miRNA-122 inhibitor via exosomes.	Momen-Heravi, 2015^173^
C57BL/6 mice	F	Liver	mirVana miRNA Isolation Kit	miRNA Microarray	miR-705 and miR-1224 increased after Lieber-DeCarli or methionine/choline-deficient (MCD) diet feeding; miR-182, miR-183, and miR-199a-3p decreased after Lieber-deCarli feeding, while MCD diet led to their increase compared to corresponding controls.	Dolganiuc, 2009^343^
Human/CH3 mice	M	Liver	Zymo RNA Extraction Kit	qRT-PCR, microRNA assay	miR-34a increase was linked to p53 decrease, and miR-483-3p decrease potentially boosted BRCA1, implicating both in Mallory-Denk body formation in AH livers.	Liu, 2015^344^
Human, C57Bl/6J mice, miR-21 knockout mice	M/F, M, M	Liver, HSCs	Arcturus PicoPure RNA isolation kit/Trizol	qRT-PCR	Depleting miR-21 inhibited NF-kappa-B activation and suppressed cytokine release; inhibition of miR-21 reduced inflammatory cytokines in HSCs during alcohol-associated liver injury; decreased VHL protein expression was observed in miR-21-expressing HSCs; treatment with anti-miR-21 significantly increased luciferase activity of the wild-type VHL 3′-UTR but not the mutant, indicating miR-21 targets VHL; miR-21-transfected HSCs showed increased NF-kappa-B phosphorylation.	Wu, 2018^89^
Wistar rats	M	Blood, liver	Trizol	qRT-PCR	In AFLD rat liver tissues, miR-181b-5p and PRMT1 were upregulated, while PIAS1 was downregulated; inhibiting miR-181b-5p, overexpressing PIAS1, or inhibiting PRMT1 improved liver function; the effects of miR181b-5p inhibition were reversed by PIAS1 silencing; PIAS1 was a target of miR-181b-5p, which regulated PRMT1 by binding to PIAS1.	Wang, 2021^92^
AML-12 hepatocyte cells, C57BL/6 mice	M	Liver, cells	Trizol	miRNA qRT-PCR SYBR	miR-217 promoted ethanol-induced fat accumulation through reduction of SIRT1 in vitro and in vivo.	Yin, 2012^345^
miR-141/200c KO mice, murine cells	M	Liver, Hepa1 cells	Trizol	qPCR	miR-141/200c deficiency normalized ethanol-mediated impairment of triglyceride secretion; miR-141/200c deficiency restored ethanol-mediated inhibition of apolipoprotein O expression and mitochondrial dysfunction.	Mostofa, 2022^346^
C57Bl/6J mice, murine cells		Liver, AML12 cells	Trizol	qRT-PCR	miR-192–5p was reduced under ethanol exposure; alcohol-induced FNDC3B expression was negatively regulated by miR-192-5p in vitro; FNDC3B inhibition led to AMPK inactivation, reduced transferrin expression, and caused iron overload and ferroptosis.	You, 2022^91^
Human cells		HepG2, HEK-293 FT	Trizol	In Silico analyses, miRNA mimic, qRT-PCR	In Silico analysis showed decreased RNA levels of ADH1A, ADH1C, ADH4, and ALDH2 in AH liver samples; miR-148a levels were lower but positively correlated with ADH4 mRNA; high alcohol doses lowered ADH4 expression, but exogenous miR-148a increased ADH4 RNA and protein levels; hsa-miR-148a-3p was a key regulator of CYP2B6 expression in AH.	Luo, 2021^347^
Sprague-Dawley rats	M	Liver	Trizol	qRT-PCR	Among dysregulated miRNAs, 17 were annotated and known to be involved in liver diseases, including miR-214, miR-741, miR-511, and miR-871-3p, targeting genes enriched in metabolic pathways such as IL-17 and TNF signaling, indicating lipid metabolism dysfunction.	Zhang, 2022^348^
Sprague-Dawley rats	M	Liver, serum	Trizol/miRNeasy Mini Kit	qRT-PCR	Alcohol increased 25 miRNAs and reduced seven miRNAs in serum (most significantly miRNA-208b-3p and miRNA-455) and increased 20 miRNAs and reduced 14 miRNA in liver tissues (most significantly miRNA-129 and miRNA-199a3p); these miRNAs are biomarkers for ASH; their dysregulation affects gene ontology biological processes and KEGG pathways.	Chen, 2013^349^
Human	M/F	Liver	miRNAprep Pure FFPE Kit	Bioinformatics tools and GEO database, qRT-PCR	Several genes and miRNAs were dysregulated in AH; miR-132, miR-92b, miR-221, and miR-222 targeted various hub genes, including SIRT1, FOXO1, CDKN1A, and BCL2L11; miR-29c potentially targeted the FOS gene.	Yao, 2019^350^
C57BL/6 mice, human cells	M	Liver, L02 cells	Trizol	qRT-PCR	Alcohol misuse increased liver miR-378b, which directly targeted/bound and reduced CaMKK2 and mediated the protein level of CaMKK2; this process was essential for lipid metabolism dysfunction leading to hepatic steatosis.	Wang, 2021^93^
Human, Ncf1-floxed mice	M/F, F	Liver	Trizol	TaqMan microRNA Reverse Transcription Kit, TaqMan miRNA Assay Kits, TaqMan Pri-miRNA Assays	Neutrophilic miR-223 expression was higher in Ncf1Lyz−/− mice with lower ROS levels compared to WT mice; in vitro, LPS, PMA, or ROS inducers decreased neutrophilic miR-223, indicating ROS inhibits miR-223 expression; this inhibition was mediated by p38 MAPK.	Ma, 2022^351^
C57BL/6 mice, mouse primary microglia	M	Brain, cells	Trizol	qRT-PCR	miR-494-3p was reduced in AH liver tissues; miR-494-3p increased the levels of alpha-SMA and fibrosis-related proteins; miR-494-3p targeted TRAF3, suppressing its expression, while TRAF3 overexpression reversed miR-494-3p’s effects.	Li, 2021^352^
Human, C57BL/6J mice, murine cells	F	Liver, AML-12 hepatocytes	Direct - zol mini RNA kit	qRT-PCR	Ethanol increased miR-150-5p in humans and mice and reduced the E3 ligase, CISH; miR-150-5p negatively regulated CISH, which then reduced ubiquitination that promoted apoptosis.	Fan, 2021^353^
Human	M/F	Blood, serum	miRNease Serum/Plasma kit	RNA sequencing	miR - 373 - 3p and miR - 6850 - 3p increased in patients who developed incident acute kidney injury; both miR - 6826 - 5p and miR - 6811 - 3p predicted incident acute kidney injury.	Tyson, 2023^354^
Human cells		Huh-7.5 cells, E47 cells		Taqman micro-RNA assays, QRT-PCR	Alcohol increased HCV RNA and miR-122 levels in Huh-7.5 cells; a miR-122 inhibitor raised cyclin G1 expression and blocked the alcohol-induced rise in HCV RNA and proteins; silencing cyclin G1 increased HCV RNA levels; the rise in miR-122 was linked to NF-kappa-B nuclear translocation and was prevented by NF-kappa-B inhibition.	Hou, 2013^355^
Human	M/F	Brain, PFC	mirVana	qRT-PCR	About 35 miRNAs in the frontal cortex of people with AUD showed small and consistent changes in expression; multiple miRNAs target genes involved in synapse formation, myelination, neurogenesis, and lipid metabolism.	Lewohl, 2011^356^
Human cells		Huh7.5 cells, Con1FL replicon cells	miRNeasy kit	QRT-PCR	Ethanol increased miR-122, which enhanced HCV replication; GW182 protein was also increased due to ethanol; silencing of GW182 in HCV cells reduced HCV RNA and miR-122 levels; use of anti-miR-122 inhibitor also reduced HCV replication.	Bukong, 2014^357^
Human	M/F	Blood	Qiazol lysis reagent	Elisa, Flow	The levels of miR-27a increased in monocytes with alcohol or alcohol+HCV as compared with HCV alone; an miR-27a inhibitor reduced alcohol- and HCV-mediated monocyte activation (CD14 and CD68 expression), polarization (CD206 and DC-SIGN expression), and IL-10 secretion in monocytes by targeting sprouty2 and enhancing ERK phosphorylation.	Saha, 2015^358^
C57Bl/6Jmice, murine cells	M	Liver, blood, AML12 cells	RNA Isolation Kit	qRT-PCR (TB Green Premix Ex Taq TM II	ASH increased SLC38A1 and miR-432 in the liver; high levels of hepatic SLC38A1 in patients with liver cancer caused lower overall survival and relapse-free survival; liver cancer patients with ASH also seemed to exhibit reduced overall survival and relapse-free survival; miRNA-432 and SLC38A1 exhibited the same expression pattern in the experiments.	Cai, 2022^90^
Human, C57BL/6J mice, human cells	M/F, M	Liver, Huh7 cells	Trizol	qRT-PCR	miR-22 reduced FGFR1 by direct targeting and decreased FGF21 by reducing the recruitment of PPAR-alpha and PGC1-alpha to their binding motifs; miR-22 inhibitor increased hepatic FGF21 and FGFR1, leading to AMPK and ERK1/2 activation; the farnesoid x receptor-agonist obeticholic acid induced FGF21 and FGFR1, as well as their inhibitor miR-22; an miR-22 inhibitor and obeticholic acid were effective in treating diet-induced steatosis, both alone and in combination.	Hu, 2020^300^
C57L/6 mice	M	Blood, liver	Direct-zol™ RNA MiniPrep	qRT-PCR	miR-122 expression in liver tissue was reduced in alcohol-fed, diethyl-nitrosamine-injected mice compared to other groups; these mice also exhibited elevated HIF1alpha mRNA levels and increased DNA binding activity of HIF-1alpha, along with higher expression of VEGFR1, indicating enhanced HIF-1alpha biological activity.	Ambade, 2016^359^
Human	M/F	Blood/Serum	Trizol	miRCURY LNA miRNA PCR assay, qRT-PCR	miR-30b-5p, miR-20a-5p, miR-146a-5p, and miR-26b-5p were reduced in both serum and liver tissues of AH patients; miR-26b-5p and miR-30b-5p inhibited RRM2 and CCND2, while miR-20a-5p inhibited CCND1 and CCND2.	Yang, 2021^360^
ICR Mice	M	Serum, liver	Trizol	qRT-PCR, Stem-Loop RT-PCR	Phenylboronic acid (150 mg/kg) markedly alleviated exosomal miR-122 and pri-miR-122 in ethanol-induced acute liver injury.	Wang, 2019^94^
Human	M/F	Blood, serum		qRT-PCR	Acute ethanol ingestion led to a significant rise in miR-122, which was small compared to the biomarker signal with acetaminophen-mediated liver injury.	McCrae, 2016^95^
Human	M/F	Blood	qEV column using miRNase	RT-PCR	Fetal alcohol exposure increased miR-199a3p, miR-214-3p, and let-7g, and reduced miR-206-3p and miR-22-2p; upregulated miRNAs caused reduced levels of stem cell regulation and differentiation.	Eguchi, 2019^96^
Sprague-Dawley rats		HepG2E47 cells	Trizol	qRT-PCR	Alcohol increased profibrotic gene expression in HSCs while accentuating cellular loss of miR19b; expression of primiR17-92 was increased in activated HSCs and in alcohol-induced liver injury; AAV2miR19b increased expression of miR19b and inhibited fibrotic gene expression and primiR17-92 cluster.	Brandon-Warner, 2016^361^
Wistar rats, human cells	M	Liver, Bel7402 cells, HEK293 cells		RT-PCR	Ethanol increased miR-214, causing decrease of glutathione reductase and POR by binding to the POR 3′-UTR region in both human and rat liver cells; thus, miR-214 mediated alcohol-induced oxidative stress by suppressing antioxidant enzymes.	Dong, 2014^97^
Swiss Webster mice	M	Liver	microRNeasy Plus kit	qRT-PCR	Twist1 was a product of quiescent HSC in adult mice, which indirectly inhibited CCN2 production through its transcriptional activation of miR-214; miR-214 suppressed CCN2 via direct binding to the CCN2 3′-UTR; during HSC activation, higher levels of CCN2 resulted at least partly from decreased Twist1 expression, which led to reduced miR-214 transcription.	Chen, 2015^98^
Wistar rats	M	Serum, liver	miRcute miRNA Isolation Kit	qPCR	miR-155 expression was increased in the liver of animals with alcohol-associated liver injury; mulberry fruit extract regulated miR-155 expression.	Qiao, 2023^362^
Sprague Dawley rats, murine cells	M	Liver, AML-12 hepatocytes		0	Alcohol reduced miR-219a-5p, which correlated with increased expression of oxidative stress / adaptor protein p66shc; protocatechuic acid increased miR-219a, causing a decrease in p66shc and ROS formation and thereby demonstrating protective effects against ALD.	Fu, 2019^363^
Sprague-Dawley rats, human, human cells	M, M/F	Liver, cells, serum, HepG2E47 cells	Trizol	qRT-PCR	Ligustrazine increased miR-145 expression and inhibited the TGF-beta/SMAD signaling pathway both in vivo and in vitro; overexpression and knockdown of miR-145 confirmed that miR-145 was involved in ligustrazine inhibition of liver fibrosis through the TGF-beta/SMAD signaling pathway.	Qiu, 2022^99^
C3H/HeOu/J mice, human	M, M/F	Liver, cells	miRNeasy kit	qRT-PCR	In ethanol-fed mice, inhibiting miR-34a increased HSC senescence but decreased senescence in the liver and hepatocytes; miR34a inhibition also lowered levels of TGFbeta1, Smad2, and Smad3; silencing miR-34a partially prevented HSC activation by LPS and enhanced HSC senescence; miR-34a inhibition reduced fibrotic gene expression in LPS-treated hepatocytes.	Wan, 2017^100^
Sprague-Dawley rats	M	Blood, liver	Trizol	qRT-PCR	miR-148a-3p expression decreased significantly in livers of alcohol-treated rats; treatment with miR-148a-3p mimics increased its expression levels; rats treated with miR-148a-3p mimics showed decreased liver indices compared to a control group after ethanol treatment, indicating a protective effect of miR-148a-3p.	Xiong, 2020^364^
C57BL/6J mice, murine cells		Liver, blood, cells, AML-12 cells	RNeasy mini kit	qRT-PCR	miR-451 was reduced in AH treatment; miR451a overexpression alleviated alcohol-induced liver inflammation and injury in AH mice by targeting HDAC8.	Du, 2020^365^
Human, C57BL/6 mice		Liver	miRNeasy Mini Kit, PicoPure RNA isolation kit	qRT-PCR	LPS increased miR-34a in macrophages, promoting M1/M2 phenotype changes and reducing Sirt1; silencing miR-34a in ethanol-treated macrophages enhanced Sirt1, reduced M1 activation by LPS, and protected against alcohol-related injury in mice.	Wan, 2023^101^

* For definitions of alcohol drinking or exposure levels used, see the original studies cited.

*Note*: 3′-UTR, 3′-untranslated region; ACLF, acute-on-chronic liver failure; AFLD, alcohol-associated fatty liver disease; AH, alcohol-associated hepatitis; ALD, alcohol-associated liver disease; ASH, alcohol-associated steatohepatitis; BMSC, bone mesenchymal stem cell; EV, extracellular vesicle; HBV, hepatitis B virus; HCC, hepatocellular carcinoma; HCV, hepatitis C virus; HEK293T, Human Embryonic Kidney 293 cell line; HSC, hepatic stellate cells; HUVEC, human umbilical vein endothelial cell; KC, Kupffer cell; LPS, lipopolysaccharide; PFC, prefrontal cortex; qRT-PCR, quantitative reverse transcription polymerase chain reaction; ROS, reactive oxygen species; RT-PCR, reverse transcription-polymerase chain reaction; WT, wildtype.

**Appendix 3:** Summary of Studies Exploring the Association Between microRNAs (miRNAs) and Alcohol Use in Neurological and Developmental Effects

Species	Sex	Sample	Key miRNA Findings*	First Author, Year
**A. Alcohol use disorder/alcohol dependence**				
Long-Evans rats	M	Blood, serum	miR30a-5p, miR195–5p, miR206–3p, and miR191–5p expression was increased by alcohol.	Ehinger, 2021^103^
C57BL/6J mice	F	Brain, mPFC	miR-411, miR-203, miR-92a, and miR-137 were reduced with alcohol exposure; antagomiR-411 infusion reduced alcohol consumption.	Most, 2019^107^
Human cells		SH SY5Y, HEK293T and 1321 N1 cells	miR-7, miR-153, miR-152, and miR-15B were differentially expressed in all three cell lines with ethanol treatment.	van Steenwyk, 2013^366^
Human, Long Evans rats	M/F	Serum, serum and neural stem cells	Differential expression of mir-92b and mir-96 was seen in serum of AUD patients; decrease of miR-92b, miR-96, miR-24, and miR-136 occurred in AUD subjects; miR-301 was reduced in serum of AUD subjects.	Ignacio, 2015^108^
Human	M/F	Saliva	hsa-miR-136-5p, hsa-miR-146b-3p were reduced, and hsa-miR-10a-5p and miR-27a-5p were increased in saliva of AUD patients.	Mead, 2022^106^
Sprague-Dawley rats	M/F	Brain, HPC	miR-125a-3p, let-7a-5p, and miR-3541 were increased in males; miR-881-3p and miR-504 were decreased in females.	Choi, 2020^112^
Human	M/F	Autopsy brain tissue, PFC	miR-130a was decreased, and miR-604 was increased in AUD cases; key miR-130a targets, including ITPR2 and ATP1A2, regulated ion channels.	Wang, 2013^116^
Rhesus macaques	M	PBMCs	Differential expression of miRNAs targeting genes involved in immune regulation and cancer pathways was found with heavy alcohol consumption.	Barr, 2016^367^
Human, murine cells	M/F	Bronchoalveolar lavage, MH-S cells	C/EBP-beta directly affected miR-1264 and miR-107 to regulate NOX1 and NOX2, respectively; pioglitazone modulated these pathways through C/EBP-beta.	Yeligar, 2021^368^
Sprague Dawley rats	M	Amygdala	Inhibition of miR-137 in adult rats led to attenuation of alcohol consumption via the two-bottle choice; rats that drank higher amounts of alcohol had a suppression of miR-137 in the amygdala.	Kyzar, 2019^113^
C57BL/B6J mice	M	Brain	Chronic ethanol exposure and single and/or repeated stress experiences reduced Bdnf mRNA expression; this was associated with increased miR-206 expression in the target brain regions (mPFC, CeA, HPC).	Solomon, 2019^369^
P rats	M	Brain	The GR-alpha suppressor, miR124–3p, was significantly increased in the NAc shell, but not the NAc core.	Alhaddad, 2020^370^
Wistar rats	Adult M only; M/F E18 Fetuses	Brain, cells	Binge drinking caused neuronal degeneration in PFC, increased oxidative stress markers, and upregulated SVCT2; miR-125a-5p downregulation and JNK/p38 MAPK activation regulated SVCT2 expression.	Tian, 2016^371^
Rhesus macaques	M	Blood	In heavy-drinking animals, miR-146a-5p, miR-17-3p, miR-17-5p, miR-21-5p, miR-150, miR125b, and miR-190 were elevated, without statistical significance; moderately drinking animals had elevated miR-155-5p, miR-17-5p, miR-29a, and miR-150, without statistical significance.	Messaoudi, 2013^109^
C57BL/6N mice	M	Dorsal HPC	The dorsal HPC level of GABAAR-delta protein, but not mRNA, was increased in high-alcohol-preferring mice and was inversely correlated to the level of miR-365–3p, suggesting an miRNA-mediated posttranscriptional mechanism contributing to elevated GABAAR-delta.	Jovasevic, 2021^118^
Human	M	Plasma	A significant relationship existed between alprazolam steady-state and miR-27b plasma concentration.	Zastrozhin, 2020^372^
C57/BL6 mice	M	Mouse alveolar macrophage cell line (MH-S)	Ethanol decreased miR-1264; miR-1264 mimic downregulated ethanol-induced Nox1 and Nox 4; miR-107 mimic downregulated ethanol-induced Nox2 and Nox 4.	Yeligar, 2023^373^
Human	M/F	Postmortem brain tissue	miR-10a-5p was increased in HPC and NAc; miR-144-3p was increased in caudate nucleus and PFC; miR-122-5p, miR-412-5p, and miR-6868-3p were reduced in amygdala, caudate nucleus, cerebellum of people with AUD.	Lim, 2021^374^
Sprague-Dawley rats, human cells		Rat striatal neurons, supraoptic nucleus region, HEK293 cells	Alcohol increased miR-9 in the supraoptic nucleus and striatum; miR-9 was identified as a mediator of posttranscriptional regulation of Ca/K+ (BK) voltage channel mRNA by binding to 3′-UTR region (increased miR-9 correlated with decreased BK mRNA).	Pietrzykowski, 2008^102^
Wistar rats	M	Brain	24 h voluntary ethanol intake was significantly reduced after lentiviral-mediated injection of let-7d into the NAc; let-7d overexpression improved motor coordination and resistance to ethanol-induced sedation; NAc D3R mRNA negatively correlated with let-7d expression.	Bahi, 2020^114^
Human	F/M	HPC	AUD was associated with higher expressions of miR-34a and miR-34c, but not of miR-34b. (Note: miR-34a participates in functioning and survival of mature neurons; miR-34b is associated with Alzheimer-like disorders; and miR-34c is implicated in the memory impairment of Alzheimer’s disease in rodents and humans.)	Santos-Bezerra 2021^375^
Rats	M	NAc	Significant changes in gene expression, in both short-term and long-term low-intensity focused ultrasound (LIFU) stimulation; immunohistochemical analysis revealed LIFU did not cause tissue damage suggesting its potential as a therapeutic approach for alcohol dependence.	Deveci, 2022^376^
Human	M/F	Saliva	Differential expression of miRNAs was observed in the saliva of alcohol-dependent individuals; salivary miRNAs predicted alcohol dependence with 79% accuracy in African Americans and 72% in European Americans; miR-10a-5p, miR1290, and miR-4499 increased, and miR-451a, miR-3613-5p, miR-7704, miR-126-3p, and miR-1273h-5p decreased, in saliva of ethanol-exposed individuals.	Rosato, 2019^119^
Female hybrid F1 mice	F	Brain, frontal cortex	Most miRNAs showed increased levels in the mouse frontal cortex similar to the PFC in people with alcohol dependence; brain genes increased in early stages of development of alcohol dependence.	Nunez, 2013^377^
Wistar rats	M	Brain tissue (PFC, HPC, corpus striatum)	miR-206 and miR-30a increased in PFC, HPC, and corpus striatum; miR-382 decreased in HPC after ethanol treatment.	Sinirlioglu, 2017^378^
Wistar Rat	M	Brain, mPFC	miRNA expression was changed with alcohol dependence in the mPFC; Gene Ontology analysis of 89 mRNA targets showed enrichment in neurotransmission, neuroadaptation, and synaptic plasticity categories.	Tapocik, 2013^115^
Human	M	Postmortem brain, PFC, NAc	Six mRNA and three miRNA modules in the NAc, and three mRNA and two miRNA modules in the PFC, were associated with alcohol dependence; significant miRNA–mRNA correlations were found (six in the NAc, four in the PFC), with miR-449a/b implicated in NAc cellular processes.	Vornholt, 2020^379^
Wistar rats, primary rat cortical neurons	M	Brain, cells	qPCR confirmed that alcohol dependence increased miR-206 expression in the mPFC, but not in the ventral tegmental area, amygdala, or NAc; virus-mediated overexpression of miR-206 in the mPFC of nondependent rats reproduced the escalation of alcohol self-administration seen following a history of dependence and significantly reduced BDNF expression.	Tapocik, 2014^104^
Human	M/F	NAc	Three miRNA modules were significantly correlated with alcohol dependence.	Mamdani, 2015^380^
Human, rats	M/F	Brain, PFC	Variants in miRNA biogenesis genes contribute to alcohol dependence risk; specific alleles in GEMIN4, AGO1, and AGO2 were significantly associated with altered alcohol dependence risk.	Gedik, 2015^381^
Wistar rats	M	Brain, PFC, plasma	Sixty-five differentially expressed miRNAs in alcohol-dependent rats; no consistency between miR-101b expression in the PFC and plasma was observed; key target genes such as PIK3CA, MAPK, NTF, BDNF, and IGF-1, suggested a regulatory network involving neurotrophic factors and protein kinases that may contribute to alcohol dependence.	Xin, 2018^382^
C57BL/6 mice	M	Limbic forebrain	miR-124 decreased in limbic forebrain after ethanol withdrawal; miR-124 increased following ethanol exposure.	Mizuo, 2012^111^
C57BL/6 mice	M	Brain	CIE resulted in changes in differential expression of miRNAs in all brain regions, with most effects on differential expression seen between the 0 hour and 8 hour timepoints.	Osterndorff-Kahanek, 2018^383^
C57BL/6 mice, primary cortical neurons		Brain, cortices	miRNAs were differentially expressed during neuronal cell maturation in culture; miR-152, miR-199a-3p, and miR-685 had the highest fold change; MeCP2 levels significantly decreased with CIE exposure and subsequent removal.	Guo, 2012^384^
Rhesus macaques	M/F	Blood/Plasma/EV	Several EV-bound miRNAs were differentially expressed in macaques with chronic drinking; miR-154 and miR-1224 targeted genes involved in blood vessel development and cell migration; miR-155 and miR-34c were linked to inflammation and immune response.	Lewis, 2022^385^
Drosophila melanogaster	F	Heads	Dysregulation of miRNA expression after alcohol exposure; miR-6 and miR-310 significantly increased ethanol sensitivity when overexpressed.	Ghezzi, 2016^386^
C57BL/6 mice	M	Cerebral cortex, midbrain	Chronic ethanol exposure increased miR-19a-3p, let7a-2-3p, and miR-214-3p, and decreased miR-488-3p, miR-384-5p, miR-193-3p, miR-214-3p, miR-201-3p, miR-3084-3p, miR-101b-3p, and miR-410-3p in cerebral cortex; CIE increased miR-3474, miR-763, and let7a-2-3p, and decreased miR-758-3p, miR-496-3p, miR-488-3p, miR-380-3p, miR-434-3p, miR-376a-3p, and miR-376b-3p in the midbrain.	Gorini, 2013^387^
Human	M/F	Blood, plasma	miR-143-3p and miR-199b-5p showed significant fold-change decreases in people with AUD; miR-143-3p/miR-146a-5p demonstrated better diagnostic performance.	Wyczechowska, 2017^388^
C57BL/6 mice	M	Primary cortical neurons	18 miRNAs in EtOHc-4D neurons and 22 miRNAs in AW8 neurons were upregulated; miR-186, miR-24, and miR-375 mimics downregulated Gabra4; miR-155, miR-186, miR-24, miR27b, and miR-375 bound to Gabra4’s 3′UTR, inhibiting protein production.	Bekdash, 2015^389^
Wistar rats	M	Brain/dorsal striatum	miR124a was downregulated upon alcohol drinking; lentiviral vectored (LV)-miR-124a silencer and LV-BDNF reduced ethanol-induced conditioned-place preference and alcohol intake.	Bahi, 2013^390^
Human	M/F	Blood	In negative phosphatidylethanol (PEth < 8 ng/mL) group, miR-133a was negatively associated with adiponectin, while miR-133a and miR-1221 together predicted adiponectin levels. In the positive phosphatidylethanol (PEth ≥ 8 ng/mL) group, miR-20a was positively associated with 2-hour glucose, and miR-20a and miR-375 predicted 2-hour glucose levels.	Bourgeois, 2022^391^
C57BL/6J mice	F	Brain	Several alcohol-induced miRNAs were correlated with alcohol consumption and their predicted mRNA synaptic targets were identified; the microRNAs in the synaptoneurosome and total homogenate responded differently to alcohol exposure, with only one common alcohol-responsive miRNA, miR-411, between the preparations.	Most, 2015^392^
Rhesus macaques	M	Primary myoblasts from skeletal muscle, plasma	SIV decreased expression of miR-1 and miR-206 in skeletal muscle; SIV decreased miR-206 expression in plasma.	Simon, 2017^393^
C57BL/6 mice, human	M	Mouse liver, human stool	Alcohol feeding upregulated miR-194 and miR-192; alcohol exposure significantly increased hepatic miR-192 levels; miR-194 suppressed the FXR gene in intestinal tissue.	Jiang, 2023^394^
C57BL/6 mice, HEK 293 cells	M	Brain regions, HEK cells	miR-30a-5p restored BDNF levels and reduced alcohol drinking levels.	Darcq, 2015^105^
Human	M/F	Serum	In alcohol withdrawal syndrome therapy, the serum concentration of BDNF increased, and the level of miR-122 decreased, in correlation to the level of depression and state anxiety.	Peregud, 2022^395^
Rhesus macaques	M/F	PBMCs	miR-181a and miR-221 were upregulated in PBMCs and miR-155 was upregulated in colon after ethanol exposure; inhibition of miR-181 and miR-221 resulted in upregulation of transcription factor (STAT3 and ARNT) protein levels and increases in growth factors (VEGF, HGF, and G-CSF).	Asquith, 2014^396^
Sprague Dawley rats	M/F	Brain, HPC	Ethanol exposure significantly altered 12 miRNAs, mostly suppressing them; only miR135a-3p was enhanced, affecting key biological processes; ethanol increased IL-6 and IkappaB-alpha expression in the hippocampus and amygdala, and reduced BDNF while increasing FGF2 expression.	Barney, 2022^397^
Human	M	Blood	People with AUD had higher prevalence of miR-146a G>C polymorphism (rs2910164) allele C; no significant differences in miR-196a2 polymorphism (rs11614913) existed between groups.	Novo-Veleiro, 2014^117^
C57BL/6 mice	M	Spleen, T cells	There was a significant reduction in T-cell miR-155 expression in mice receiving ethanol combined with burn injury compared to those receiving sham injury; miR-155−/− mice did not show any change in T cell release of IFN-gamma or expression of nuclear factors compared to wildtype mice.	Li, 2014^398^
C57BL/6 mice	M	Small intestine epithelial cells	Eleven miRNAs were increased, and six miRNAs were decreased following alcohol and burn injury; differentially expressed miRNAs were involved in signal transduction pathways (Foxo, MAPK, PI3K, AKT, TNF, HIF-1, JAK-STAT), endocytosis, focal adhesion, actin cytoskeleton regulation, ubiquitin proteolysis, and the cell cycle.	Herrnreiter, 2021^399^
UChB rats	M	Plasma, cerebellum	In cerebellar tissue, expression of miRNAs155-5p, miR-146a-5p, miR-126-3p, and miR-132-3p was increased in the ethanol group and reduced in the ethanol+caffeine group; in plasma, caffeine significantly elevated miR-126-3p and miR-132-3p levels and decreased miR-155-5p levels.	Rossetto, 2019^110^
**B. Neuroinflammation and neurobiology**				
Human, C57BL/6 mice, TLR4-KO	M/F	Blood, plasma, cerebral cortex, liver	Anti-inflammatory miRNAs (miR-146a-5p, miR-21-5p, and miR-182-5p) were downregulated in females and upregulated in males following ethanol treatment.	Ibáñez, 2020^400^
Wistar rats	M	Brain, NAc	Ethanol significantly increased miR-155 and decreased miR-let7b, miR-96, and miR-182 in NAc; this dysregulation affected TLR signaling (TLR4 and TLR7).	Airapetov, 2023^120^
C57BL/6 mice		Microglial cells, cerebral cortex	Ethanol increased miR-339-5p in microglial cells; miR-339-5p inhibited alcohol-stimulated NF-kappa-B pathway and its downstream targets IL-1B, IL-6, TNF-alpha; a miR-339-5p inhibitor stimulated the NF-kappa-B pathway.	Zhang, 2014^122^
C57BL/6 mice (WT and TLR4−/−)	F	Brain, cerebral cortex	mmu-miR-183, mmu-miR-143, and mmu-miR-96 were decreased, and mmu-miR-351, mmu-miR-150, mmu-miR-125b, mmu-miR-1981, mmu-miR-7224, and mmu-let-7b were increased after alcohol treatment; alcohol had less influence on miRNA expression in mice lacking the TLR4 receptor.	Ureña-Peralta, 2018^123^
Murine cells, B6.Cg-Tg(Thy1-YFP)HJrs/J mice, C57BL/6 mice	F	Primary cortical astrocytes, brain	Treatment with bisdemethoxycurcumin-bearing nanoconjugate reduced the levels of inflammatory proteins and miRNAs associated with inflammation and TLR4 signaling in mouse cerebral cortex.	Cuesta, 2021^124^
C57BL/6, TLR4-Knock-out (GD17) mice	M/F	Brain	Ethanol exposure increased the secretion of EVs and inflammatory-related proteins (TLR4, NF-kappa-B-p65, IL-1R, caspase-1, NLRP3); TLR4-knockout astrocyte-derived EVs could be internalized by naïve cortical neurons to increase the neuronal levels of inflammatory protein (COX-2) and miRNAs (e.g., mir-146a) and compromise their survival.	Ibáñez, 2019^125^
C57BL/6J mice	F	Blood, brain	Chronic ethanol feeding increased miR-155 and miR-132 in mouse cerebellum; miR-155 deficiency protected mice from alcohol-induced inflammatory cytokines and reduced TNF-alpha, MCP1, pro-IL-1beta, and pro-caspase-1 levels; alcohol-fed TLR4-KO mice show reduced miR-155, NF-kappa-B activation, and neuroinflammation compared to controls.	Lippai, 2013^121^
Human neuroblastoma cells	M/F	SH-SY5Y cells/IMR-32 human neuroblastoma cells	Ethanol increased miR-497 and miR-302b, and decreased BCL2 and/or cyclin D2 in cells; overexpression of only miR-497 increased caspase-3/ROS–mediated apoptosis and necrosis and induced ROS generation, mitochondrial membrane potential disruption, and cytochrome c release.	Yadav, 2011^401^
Sprague - Dawley rats	M	Brain, amygdaloid tissue	miR-494 and miR-130a decreased significantly, while miR-191 increased significantly with ethanol exposure; mRNA levels of chromatin remodeling genes (Cited2, CBP, and p300) in the amygdala were higher after ethanol exposure, suggesting involvement of CREB pathways.	Teppen, 2016^402^
Sprague-Dawley rats	M	Brain, NAc	miR-382 was reduced in NAc following ethanol exposure; miR-382 attenuated upregulation of DRD1 and DeltaFosB transcription factors.	Li, 2013^403^
Human	M/F	Postmortem brain, frontal cortex	Twelve miRNAs, including a notable cluster on chromosome 14q32 (miR-377, miR-379), were up-regulated in the brain of people with AUD; overexpression of miRNAs in people with AUD suppressed mRNAs for oligodendrocyte proliferation and brain functions.	Manzardo, 2013^404^
Human cells		HEK293T cells	miR-7, miR-203, and miR-144 regulated specific 14-3-3 protein isoforms; miR-203 selectively downregulated 14-3-3-theta, while miR-7 and miR-144 up-regulated 14-3-3-gamma.	Mathew, 2016^405^
Rat		PC12 cell line	An miR-96-5p inhibitor induced apoptosis and increased the expression of TAp73 in PC12 cells.	Yang, 2023^406^
Wistar rats	M	Brain, HPC, plasma	Mid/peri-pubertal binge ethanol exposure reduced miR-26a and miR-495 in dorsal HPC and induced dysregulation of miR-10a and miR-495 in ventral HPC; Drosha/Dicer enzymes altered expression with long term ethanol exposure; BDNF and SIRT1 genes were differentially expressed, causing disruption in maturation of HPC during puberty.	Prins, 2014^407^
Human	M/F	HPC tissue	Ethanol increased TLR7 activation and release of HMGB1-miR-let-7 complexes in microglia-derived vesicles that caused neurotoxicity via TLR7 activation.	Coleman, 2017^177^
miR-21-lacZ mice, human	M	Brain	miR-21 was highly expressed in adult oligodendrocytes in the white matter and myelinated portions of the gray matter.	Miguel-Hidalgo, 2017^408^
Human	M/F	Blood	In heavy drinkers, higher expression of miR-10a and miR-21 after stress, which correlated with increased alcohol consumption, whereas in moderate drinkers, these miRNA expressions were not statistically significant, and no correlation was observed; miR-21 induction was closely linked to adrenocorticotropic hormone induction.	Beech, 2014^409^
Cynomolgus macaques	M	PFC area 46	Animals that drank alcohol exhibited 567 upregulated and 675 downregulated DEGs; miRNA target analysis showed upregulated genes were enriched in target sites for 197 human miRNAs; highly connected miRNAs target genes included DNAJB4, KLF10, and PHF6.	Walter, 2020^410^
Human	M/F	Blood	The hsa-miR-4456–associated methylation locus cg01299774 was differentially methylated in alcohol dependence, suggesting that it may be primarily associated with the addictive component observed in hypersexual disorder.	Boström, 2020^411^
**C. Fetal alcohol spectrum disorder/neurodevelopmental disorder**				
Sheep	F	Blood	miR-9, miR-15b, miR-19b, and miR-20a were identified as biomarkers of ethanol exposure in both pregnant ewes and newborn lambs.	Balaraman, 2014^129^
Human embryonic stem cells		hESC	miR-145 mediated alcohol toxicity by reducing Sox-2 and ERK expression, depleting neural progenitors.	Louis, 2017^130^
C57BL/6 mice		Cerebellum, primary culture of cerebellar granule neurons	miR-29b regulated ethanol-induced cerebellar neuron apoptosis via SP1/RAX/PKR cascade.	Qi, 2014^131^
Human	M/F	Blood, serum	miRs-122-3p, miR-126, miR-216b, miR-221-5p, miR-3119, miR-3942-5p, miR-4704-3p, miR-4743, miR-514b-5p, and miR-602 were the top 10 discriminators between groups with and without PAE.	Gardiner, 2016^412^
Zebra fish		Embryo tissue	35 miRNA transcripts were significantly differentially expressed; suppressing miR-153c during development affected the formation of craniofacial skeletal structures.	Tal, 2012^413^
Human	F	Blood, plasma	Dysregulation of miRNAs in response to PAE was dependent on fetal sex.	Salem, 2020^414^
Zebra fish		Embryo	Ethanol significantly upregulated miR-153a, miR-725, miR-30d, let-7k, miR-100, miR-738, and miR-732.	Soares, 2012^415^
C57BL/6 mice		Fetal mouse cerebral cortical neural precursors	Ethanol suppressed the expression of ethanol-sensitive miRNAs (miR-9, miR-21, miR-153, and miR-335) and a nicotine-sensitive miRNA (miR-140-3p), while nicotine antagonized this suppression at physiologically relevant concentrations; nicotine upregulated nAChR subunit mRNAs, and the effects were blocked by the nAChR antagonist mecamylamine.	Balaraman, 2012^208^
Sprague-Dawley rats	M/F	Brain, HPC	Ethanol exposure increased miRNA variance, but this was attenuated by choline; ethanol raised miR-200c expression in late gestation, and choline reduced it.	Balaraman, 2017^416^
NSCs from C57BL/6 mouse fetuses	M/F	Neurospheres, EVs	Ethanol changed the miRNA profile in NSC-derived EVs; miR-140–3p overexpression increased NSC proliferation by modulating the cell cycle.	Tseng, 2019^176^
Long Evans rats	M/F	Brain, amygdala, ventral striatum	PAE disrupted brain miRNA and mRNA networks, but social enrichment reversed ethanol-induced changes in specific miRNAs (miR-204, miR-299a, miR-384-5p, miR-222-3p, miR301b-3p, miR-6239); p53, CREB, glutamate, and GABA signaling were most affected pathways.	Ignacio, 2014^417^
Sprague–Dawley rats, pups	M	Brain, ventral tegmental area	Nicotine-alcohol perinatal exposure increased 64 unique miRNAs and decreased 67 miRNAs in dopaminergic neurons; while in non-dopaminergic neurons, 46 miRNAs were increased and 217 miRNAs were decreased.	Kazemi, 2021^418^
Human	M/F	Plasma	At 2 weeks after birth, hsa-miR-421 was increased and hsa-miR-193a-3p and hsa-miR-92a3p were decreased in plasma of infants exposed to ethanol; at 6.5 months, hsa-miR-30a-5p and hsa-miR-193b-3p were increased.	Mahnke, 2021^419^
C57BL/6J mice	M	HPC, serum	Ethanol increased miR-135a, miR-135b, miR-467b-5p, and miR-487b in HPC; miR-467b-5p targeted VGLUT2; ethanol increased miR-135a, miR-135b, and miR467b-5p in serum.	Zhang, 2015^420^
Human	F	Plasma	Alcohol consumption caused dysregulation of maternal plasma miRNA levels; 11 miRNAs were significantly elevated in the plasma of the heavily prenatally exposed and affected group compared to the heavily exposed but apparently unaffected and unexposed groups.	Balaraman, 2016^180^
Murine cells		NCCs (Joma1.3 cells)	Ethanol significantly increased miR-34a expression in NCCs; ethanol induced inhibition of neural differentiation of NCCs mediated by miR-34a.	Fan, 2019^421^
C57BL/6J mice		Blood, fetal brain	miR-10a, miR-10b, and Hoxd3, Hoxd4, Hoxb4, Hoxb5 genes were significantly increased in ethanol-exposed fetal brains; folic acid supplementation was able to counteract the ethanol-induced upregulation of miR-10a.	Wang, 2009^422^
Sprague - Dawley rats	M	Brain, ventral tegmental area	Differential miRNA expression was observed following perinatal alcohol and nicotine+alcohol exposure; miR-30b targeted Gnai2/Cotl1 after alcohol and Gnai2/Bnip3l after nicotine+alcohol; miR-26b targeted Nxpe3 after nicotine+alcohol vs. alcohol.	Kazemi, 2020^423^
C57BL/6J mice	M/F	Brain microvascular endothelial cells, cortex	Increase of miR-150-5p during PAE caused decreased angiogenic factor Vezf1 expression, which inhibited migration/tube formation of brain microvascular endothelial cells.	Perales, 2022^424^
Mice (type not specified)	M/F	Cortex, corpus callosum	miR-17-5p, which was downregulated by PAE, repressed Epha4 mRNA in neocortical callosal projection neurons in establishing interhemispheric connectivity.	Altounian, 2023^425^
Zebrafish, murine NCCs		NCCs (Joma1.3 cells), zebrafish embryos	Ethanol reduced miR-135a in NCCs and zebrafish embryos; Siah1 was a direct target of miR-135a; miRNA-135a overexpression reduced ethanol-induced p53 phosphorylation and PUMA/Bak expression.	Yuan, 2020^426^
Wistar rats	M	Brain, HPC, plasma	Ethanol exposure downregulated miR-19a-3p, miR-19b-3p, miR-29a-3p, miR-29c-3p, miR34a, and miR-488-3p in ventral HPC of male rats during peripuberty stage; dysregulation of miRNAs had an effect on BDNF, which is important for development processes in ventral HPC.	Asimes, 2019^427^
NCCs, C57BL/6J mice	F	Cells, embryo	miR-125b was deregulated in ethanol-exposed NCCs and mouse embryos, which contributed to ethanol-induced apoptosis in NCCs and subsequent embryotoxicity by upregulation of the proapoptotic proteins, Bak1 and PUMA.	Chen, 2015^428^
Zebrafish, C57BL/6/SJ mice		Zebrafish embryos, mouse NSCs	Ethanol transiently suppressed miR-9, as well as FGFR-1 and FOXP2; alterations in miR-9 expression were correlated with severity of ethanol-induced teratology.	Pappalardo-Carter, 2013^134^
POMC-EGFP mice, murine cells	M	Brain, mediobasal hypothalamus, mHypoA-POMC/GFP cell line	Ethanol exposure increased miR-383 and miR-384, binding to POMC 3′-UTR; inhibitors restored POMC mRNA/protein levels, while mimics reduced POMC mRNA.	Gangisetty, 2023^145^
C57BL/6J mice	M/F	Brain	Selective alterations in DNA methylation and miRNA expression were found in adults that were exposed to alcohol during neurodevelopment.	Laufer, 2013^429^
Wistar rats		Fetal cartilage	miR-200b-3p was strongly expressed following ethanol exposure; miR-200b-3p inhibitor reversed the inhibitory effects of ethanol.	Ni, 2022^430^
C57BL/6J mice, rhesus macaques	M/F	Labyrinth, junctional, and decidual zone of placenta	PAE inhibited placental pro-epithelial mesenchymal transformation pathways in rodents and primates; 11 maternal circulating miRNAs collectively mediated this effect, affecting cell proliferation, epithelial mesenchymal transformation pathways, inducing cell stress, and causing abnormal endocrine maturation in trophoblasts.	Tseng, 2019^431^
C57BL/6 mice	M/F	Neural stem cells	miR-153 overexpression prevented the effects of ethanol exposure on mRNA targets of miR-153.	Tsai, 2014^432^
Vervet monkeys	M	Brain	Several differentially expressed miRNAs were found in a non-human primate model of PAE; notably, miR-9-5p was among the upregulated miRNAs.	Gillis, 2023^136^
Wistar rats	M/F	HPC	Ethanol exposure of developing brain tissue led to increased miR-137 and miR-501-3p expression that downregulated AMPA synaptic transmission, a process mediated by mGlu5 in immature organotypic HPC slices.	Gerace, 2023^433^
C57BL/6J mice	M	Blood, reproductive tract, liver, spleen	Chronic preconception alcohol exposure in mice shifted sperm RNA composition, with a decrease in transfer RNA-derived small RNAs and an increase in piwi-interacting RNAs; miR-21 and miR-30 were enriched, while miR-142 was reduced.	Bedi, 2019^434^
Zebrafish, human cells		Human NCCs and human placode cells	Ethanol increased miR-126 in neural crest cell exosomes, which led to a decrease in SDF1 expression that disrupted NCC migration; ethanol disrupted migration of NCCs and placodal cells, but exosomes derived from ethanol-treated NCCs could mediate this disruption.	Li, 2023^435^
Zebrafish	M/F	NCCs (Joma1.3 cells)	Ethanol exposure of NCCs significantly increased miR-34a expression; downregulation of miR-34a prevented ethanol-induced reduction of *Snail1* in NCCs; miR-34a inhibition prevented ethanol-induced inhibition of NCC migration.	Fan, 2022^436^
C57BL/6 mice		Mouse whole brain	miR-26b increase following ethanol exposure correlated with reduced Cnr1 transcript in adult mice that were neurodevelopmentally exposed to ethanol.	Stringer, 2013^437^
C57BL/6 mice		Fetal NSCs	Ethanol exposure increased the pre-miR-9-2 coding region.	Burrowes, 2017^139^
C57BL/6NHsd	F	Placenta	Maternal miRNAs gestationally elevated due to heavy exposure to alcohol (HEamiRNAs) reduced umbilical cord blood flow during gestation.	Pinson, 2023^179^
C57BL/6 mice	M/F	Brain	In the ethanol exposed fetal brain (short-term effect), 63% of DEGs were upregulated compared to 40% in the adult brain (long-term group); ingenuity pathway analysis identified six miRNAs, including miR-146b, miR-208b, miR-302c, miR-335, miR-449, and miR-455; all of these, except miR-302c, showed downregulation.	Mantha, 2014^438^
Human	F	Plasma, brain	Ethanol downregulated miRNA-9 in vivo in both brain and fetal central nervous system-derived EVs.	Goetzl, 2019^137^
C57BL/6J mice	M/F	Brain	Ethanol exposure during the third-trimester equivalent had pervasive consequences that broadly affected synaptic communication, altering HPA axis function and higher cognitive functioning.	Kleiber, 2014^439^
Fisher-344 strain rats	F	Blood	Fetal alcohol exposure programmed the pituitary to upregulate miR-9 expression, targeting 3′-UTR of D2r gene, reducing its expression, and promoting increased prolactin production and secretion.	Gangisetty, 2017^135^
Wistar rats	F	Whole brain	Ethanol exposure resulted in a decrease in miR-355 and miR-21 in the PFC.	Labib, 2021^440^
Fetal mouse cerebral cortical neurospheres	M/F	Cortical neuropsheres	Ethanol (320mg/dL) suppressed miR-21 and miR-335; miR-21 suppression involved GABAA receptors, while miR-335 suppression did not; miR-21 was antiapoptotic, whereas miR-335 was proapoptotic; knocking down miR-335, miR-21, and miR-153 increased Jagged-1 mRNA.	Sathyan, 2007^41^
Sprague - Dawley rats	F	Amniotic fluid, EVs	Functional roles were identified for amniotic fluid exosomal miRNAs (miR-199a-3p, miR-214-3p and let-7g).	Tavanasefat, 2020^441^

* For definitions of alcohol drinking or exposure levels used, see the original studies cited.

*Note*: 3′-UTR, 3′-untranslated region; AUD, alcohol use disorder; BDMC, bisdemethoxycurcumin; BDNF, brain-derived neurotrophic factor; CIE, chronic intermittent ethanol exposure; CeA, central nucleus of the amygdala; DEG, differentially expressed gene; EV, extracellular vesicle; HPC, hippocampus; mPRC, medial prefrontal cortex; NAc, nucleus accumbens; NCC, neural crest cell; NSC, neural stem cell; PAE, prenatal alcohol exposure; PBMC, peripheral blood mononuclear cell; PFC, prefrontal cortex; POMC, proopiomelanocortin; qRT-PCR, quantitative reverse transcription polymerase chain reaction; ROS, reactive oxygen species.

**Appendix 4:** Summary of Studies Exploring the Association Between Alcohol Use and Long Noncoding RNAs (lncRNAs)

Species	Sex	Sample	RNA Extraction Method	Method of lncRNA Analysis	Key lncRNA Findings*	First Author, Year
C57BL/6 mice, murine cells	M	Liver, AML-12 cells	Trizol	qRT-PCR/RIP	lncRNA_AIRN increased in AFL, lncRNA_AIRN silencing activated other pathways that reduced lipid accumulation and may be used as a therapeutic for AFL.	Shen, 2021^442^
Human	M	Peripheral blood cells	GoldMag blood DNA Kit	Genotyping Agena MassARRAY	lncRNA MIR31HG showed increased correlation to osteonecrosis of the femoral head with alcohol exposure.	Liu, 2022^443^
C57BL/6 mice	M	Liver	Trizol	qRT-PCR/RIP	Increase in LINC01093 could inhibit hepatocyte apoptosis in AH.	Shi, 2019^444^
Human		Liver biopsies	Pinpoint Slide RNA isolation System II	RNA sequencing	168 lncRNAs were significantly dysregulated in livers of people with AH.	Zhong, 2020^445^
Human		Esophageal epithelial tissues	Trizol	qRT-PCR	LINC01133 was affected by drinking status and could influence esophageal squamous cell carcinoma.	Yang, 2018^446^
Human	M/F	Esophageal squamous cells	Trizol Tiangen	RNA seq/qRT-PCR/RNAi	LINC00707 expression was increased in people with esophageal cancer who drink alcohol.	Gao, 2023^447^
Human		Femoral heads, blood cells	Trizol	qRT-PCR	An array of lncRNAs were identified that could be involved in important pathways and could be used as biomarkers in alcohol-induced osteonecrosis of the femoral head.	Li, 2021^448^
Human	M	Brain	miRNeasy mini-Kit	Arraystar m6AmRNA & lncRNA Epitranscriptomic Microarray Assay	29 mRNAs, five lncRNAs, and three miRNAs were differentially methylated (|FC| ≥ 2 and p < 0.05) in people with AUD.	Liu, 2022^449^
Human	M/F	FFPE esophageal cancer tissue	NucleoSpin total RNA FFPE Kit	qRT-PCR	lncRNA-UCA1 expression was substantially correlated to alcohol drinking.	Aalijahan, 2020^450^
Human, C57BL/6 mice, murine cells	M/F	Blood, serum, liver, AML-12 cells	Trizol	siRNA, qRT-PCR	NEAT1 and SOCS2 were highly expressed while miR-129-5p was poorly expressed in serum of people with ASH and mouse models; inhibiting NEAT1 or elevating miR-129-5p alleviated liver pathology and hepatocyte apoptosis.	Ye, 2020^194^
Human	M/F	Postmortem brain	mirVana miRNA Isolation Kit	RNA sequencing	The lncRNA MALAT increased in AUD brains and interacted with splicing factors, leading to the conclusion that AUD affects lncRNAs that are functionally related to splicing.	Van Booven, 2021^200^
Wistar rats	M	Brain	miRNAEasy mini kit	qRT-PCR	Changes in the lncRNA Lrap created increases in alcohol consumption and preference; Lrap was also had a potential role in alternative splicing.	Saba, 2021^451^
C57BL/6 mice, murine cells	M	Liver, AML-12 cells	Trizol	qRT-PCR/RIP	lncRNA 1700020I14Rik increased in AH.	Wu, 2022^452^
C57BL/6 mice, human cells	M	Liver, LX-2 cells	Trizol	qTR-PCR	XIST overexpression increased protein levels of alpha-SMA and CoL1A1; luciferase activity in XIST suggested direct binding between XIST and miR-29b.	Xie, 2019^246^
Mouse cell line		Neuro2a cells	Trizol	qRT-PCR	Ethanol exposure increased lncRNAs Rnu3a and 4930507D05Rik and downregulated Tbrg3, Kcnq1ot1, Tug1, and Xist.	Choi, 2022^247^
SD and BN rats	M/F	Brain	Trizol	RT-PCR	Rat Dio3os transcript did not obviously overlap the rDio3 transcript itself but was imprinted and coregulated with rDio3 both at the level of total expression and of imprinted expression; this represented the first example of paired sense/OS transcripts arising from the same allele.	Dietz, 2012^453^
C57BL/6 mice	M/F	Left hippocampus	Trizol	RT-PCR	Female Pitt1, Pitt3, and Pitt4 mutants showed reduced ethanol intake in EOD-2BC assay; Pitt1 and Pitt2 males exhibited altered ethanol preference; Pitt3 and Pitt4 females had increased ethanol preference.	Plasil, 2022^454^
C57BL/6 mice, murine cells	M	Mouse hepatocytes, AML-12 cells	Trizol	miRNA qRT-PCR Detection Kit	miR-let-7c-5p overexpression inhibited ethanol-induced hepatic steatosis and apoptosis; chronic ethanol led to an induction of lncRNA MEG3 expression.	Wang, 2018^455^
Human	M/F	Postmortem amygdala tissue		qRT-PCR	BDNF-AS was upregulated due to decreased RNA methylation in the amygdala in people with AUD.	Bohnsack, 2019^190^
C57BL/6 mice		Neural stem cells	miRNeasy Mini Kit, Trizol	qRT-PCR, RNA sequencing	Ethanol led to a dose-related increase of mOct4pg9.	Salem, 2021^212^
C57BL/6J mice, murine cells	M	Liver, AML-12 cells	Trizol	qRT-PCR	Ethanol caused verified differential expression of five lncRNAs in the liver (increased lmmu_lnc_1700023H06Rik, mmu_lnc_0610005C13Rik, mmu_lnc_Gm12265 and decreased mmu_lnc_AW495222, mmu_lnc_Gm45724); these lncRNAs were involved in pathways such as lipid metabolism, inflammation, and oxidative stress.	Dou, 2021^456^
Sprague-Dawley rats	M	BMSCs	Trizol	qRT-PCR	lnc-HOTAIR/miR-122/PPAR-gamma signaling was found to mediate alcohol-induced osteonecrosis of the femoral head in rats; ethanol inhibited miR-122 expression by promoting lnc-HOTAIR.	Le, 2023^211^
C57BL/6 mice	M	Mouse primary hepatocytes	Trizol	qPCR	lncRNA Gm5091 was downregulated in hepatocytes of mouse during alcohol-associated hepatic ﬁbrosis (AHF), and alcohol-treated primary hepatocytes; lncRNA Gm5091 negatively regulated cell migration, reactive oxygen species levels, Il-1beta secretion, collagen I expression, and hepatocytes activation markers; Gm5091 bound miR-27b, miR-23b, and miR-24, reducing their levels and alleviating AHF in mice.	Zhou, 2018^457^
Human, human cells	M/F	Serum, L02 cells	Trizol	RT-PCR	The expression of UCA1 was increased in serum of patients with AFL and in ethanol-induced L02 cells. Knockdown of UCA1 reversed the inhibiting effect of ethanol on the biological behavior of L02 cells, including cell proliferation, migration, and apoptosis.	Xiang, 2022^216^
Sprague–Dawley rats		Serum	Trizol	qRT-PCR	lncRNA HOTAIR targeted miR-148a-3p to activate S1PR1 expression, which ultimately supported the proliferation and colony formation while inhibiting apoptosis of hepatic stellate cells in AH rats.	Chen, 2023^209^
Human		Postmortem brain, NAc	mirVana - PARIS kit	Arraystar Human lncRNA Array v3.0	19 lncRNAs and 5 protein-coding gene modules, which were linked to alcohol dependence, were enriched in neuronal and immune-related functions; coexpression analysis revealed thousands of correlations between lncRNA and protein-coding gene hubs, suggesting potentially regulatory functions of lncRNAs in alcohol dependence.	Drake, 2020^458^
Human, L02, Hep3B cells		The Cancer Genome Atlas datasets, liver	SurePrep RNA Isolation kit	qRT-PCR	Databases revealed significant differential expression of lncRNAs due to hepatitis virus and in tumor samples; lnc-CFP-1:1 and lnc-CD164L2-1:1 were downregulated in people with hepatocellular carcinoma who consumed alcohol and were significantly downregulated in both cell lines.	Zheng, 2018^459^
Human		Liver, blood	QIAamp RNA Blood Mini Kit	Arraystar human lncRNA microarray version 3.0/qRT-PCR	Differential expression of lncRNA in alcohol-associated cirrhosis patients compared to healthy controls, with AK128652 and AK054921 being among the most highly expressed in both plasma and liver tissue of alcohol-associated cirrhosis patients. AK054921 and AK128652 could be promising biomarkers for predicting mortality in people with alcohol-associated liver disease.	Yang, 2017^217^
Human	M/F	Intestinal epithelial cells	Genome Analyzer IIx	miRNA-seq	lnc-PSD4-1 and lnc-NETO-1 were differentially expressed due to alcohol consumption, based on RNA-seq analysis of clinical data; low expression of the lnc-PSD4-1 isoform, lncPSD4-1:14, exhibited a strong correlation with high survival rates in a Cox proportional hazards regression model.	Yu, 2016^460^
Human	M/F	Database	University of California Santa Cruz Xena server		Four lncRNAs (AC012640.1, AC013451.2, AC062004.1, and LINC02334) were used to construct a risk assessment model to predict overall survival for patients with alcohol-related hepatocellular carcinoma, and five lncRNAs (ERVH48-1, LINC02043, LINC01605, AC062004.1, and AL139385) were used to predict recurrence-free survival.	Luo, 2020^461^
C57BL/6J mice	M	Brain	miRNeasy Mini Kit	qPCR, RNA seq	Ethanol affected the attachment of 107 lncRNAs, while the addition of PARP inhibitor ABT-888 reduced this effect, altering only 60 lncRNAs; lncRNAs GAS5, 5430416N02Rik, and 1110038B12Rik play roles in chromatin remodeling, gene regulation, and protein degradation, while Snhg family members function in oncogenic pathways and DNA damage response.	Rizavi, 2023^462^
Human, rat	F/M	Brain	Guanidinium thiocyanate, phenol-chloroform method	PCR/Northern blot	In people with AUD, MALAT-1 lncRNA was greatly upregulated in the cerebellum, brainstem, and hippocampus; the strong increase in MALAT-1 was not seen in rats but significant increase in MALAT-1 in cortex occurred 24 hours after withdrawal.	Kryger, 2012^191^
Human	M/F	Blood	Qiamp RNA Blood mini kit	qRT-PCR	Patients with esophageal squamous cell carcinoma with a history of alcohol consumption had downregulation of EWSAT1 expression.	Uttam, 2024^218^
C57BL/6J mice	M/F	Brain	SeraMir EV RNA Purification Kit	Mouse LncRNA Array v4.0	Chronic intermittent ethanol vapor exposure significantly changed the RNA cargo of brain-derived EVs, which could impact neuronal function; sex differences existed in RNA cargo of EVs following chronic ethanol exposure; lncRNA modules FM_8 and MM_5 were downregulated in EV in males and females after chronic intermittent ethanol exposure.	Baratta, 2022^463^
Human	M/F	Blood		Whole genome sequencing	Alcohol consumption reduced LINC02347; variants upstream of LINC02347, such as rs4309206, showed significant association with alcohol-induced depression.	Peng, 2019^464^
Human	M/F	Blood	Trizol	qRT-PCR	Serum lincRNA-p21 level increased in patients with hepatitis virus infection, hepatitis B cirrhosis, and hepatitis B virus-related hepatocellular carcinoma, compared with healthy controls.	Wang, 2019^465^
BalB/c mice	M	Serum, pancreas, liver, brain, spleen, lung, heart	GEO deta base/Robust Multi-Array Average	qRT-PCR	Alcohol increased lncRNA CRNDE in mice with alcohol-associated liver disease, which facilitated apoptosis via extrinsic and intrinsic apoptotic pathways; CRNDE was also significantly involved with MAPK and Wnt pathways.	Yan, 2021^466^

* For definitions of alcohol drinking or exposure levels used, see the original studies cited.

*Note*: AUD, alcohol use disorder; AFL, alcohol-associated fatty liver; AH, alcohol-associated hepatitis; ASH, alcohol-associated steatohepatitis; AHF, alcoholic hepatic fibrosis; EV, extracellular vesicles; FFPE, formalin-fixed paraffin embedded; NAc, nucleus accumbens; qRT-PCR, quantitative reverse transcription polymerase chain reaction; qPCR, quantitative polymerase chain reaction; RT-PCR, reverse transcription polymerase chain reaction; RIP, RNA immunoprecipitation.

**Appendix 5:** Summary of Studies Exploring the Association Between Alcohol Use and Circular Noncoding RNAs (circRNAs)

Species	Sex	Sample	RNA Extraction Method	Methods of circRNA Analysis	Key circRNA Findings*	First Author, Year
Human	M/F	Serum, exosome	Trizol	circRNA sequencing, qRT-PCR	Differentially expressed miRNA was found in people with alcohol dependence; hsa_circ_0004771 was related to severity of alcohol dependence.	Liu, 2021^259^
C57BL/6J mice	M	Brain, blood	Trizol	circRNA sequencing, qRT-PCR	CircRNA differential expression in brain and blood of mice with chronic intermittent ethanol exposure was linked to addiction-related neurotransmitter and signal transduction pathway changes (from KEGG pathway analysis).	Gong, 2022^260^
C57BL/6J mice	M/F	Brain	miRNeasy RNA isolation kit	qRT-PCR	circHomer1 was reduced in male adults and lncRNA H19 was increased in male adults with PAE.	Papageorgiou, 2023^243^
C57BL/6J mice	M/F	Brain	miRNeasy RNA isolation kit	circRNA Microarray, qRT-PCR	circSatb2 was increased in PAE males and circPtchd2 was reduced in PAE male pups.	Paudel, 2020^256^
C57BL/6J mice	M	Liver	Trizol	qRT-PCR	mou_circ_1657 was increased in the liver of mice with alcohol-associated liver disease, which inhibited miRNA-96-5p.	Dou, 2020^258^
Human	M	Brain, frozen nucleus accumbens	mirVANA-PARIS kit	circRNA Array, Affymetrix GeneChip miRNA 3.0 Array	circRNA-406742 and miR-1200 showed significant interaction.	Vornholt, 2021^262^
C57BL/6J mice	M	Liver	Total RNA Sample Preparation kit	circRNA sequencing, qRT-PCR	Knockdown of mm9_circ_018725 reduced hepatocyte apoptosis in Aml-12 cells.	Meng, 2019^261^
Long-Evans rats	F	Blood, spinal cord tissues	miRNeasy RNA isolation kit	circNA RNA Microarray	circVopp1 was significantly decreased in blood leukocytes in PAE, spinal circVopp1 was increased in PAE; spinal circItch and circRps6ka3 were reduced in PAE.	Noor, 2023^255^
C57BL/6J mice	M	Liver, Kupffer cells	Trizol	qRT-PCR	Circ_1639 was increased in Kupffer cells in ethanol-induced liver injury model; circ_1639 targeted miR-122 (sponge).	Lu, 2019^257^
Human		Blood, peripheral blood mononuclear cells	Trizol	qRT-PCR	hsa_circHERPUD2 expression was negatively impacted by alcohol.	He, 2023^467^

* For definitions of alcohol drinking or exposure levels used, see the original studies cited.

*Note*: PAE, prenatal alcohol exposure; qRT-PCR quantitative reverse transcription polymerase chain reaction.
