# Correction: “Noncoding RNA and Alcohol Use Disorder: A Scoping Review of Current Research and Knowledge Gaps”

**DOI:** 10.35946/arcr.v46.1.04

**Published:** 2026-06-18

**Authors:** Deepa Upreti, Rosaline P. Kumar, Justin B.J. Chen, Sneha L. Sonti, Abigail V. Bowring, Sheila W. Green, Rajesh C. Miranda

This is a correction to: Alcohol Res. 2025; 45(1):06. https://doi.org/10.35946/arcr.v45.1.06.

An error appeared in Appendix 4. The text in Row 23 (Zhou, 2018) under column 6 (Key lncRNA Findings) of that table reads: “lncRNA Gm5901 was downregulated in hepatocytes of mouse during alcohol-associated hepatic ﬁbrosis (AHF), and alcohol-treated primary hepatocytes; lncRNA Gm5901 negatively regulated cell migration, reactive oxygen species levels, Il-1beta secretion, collagen I expression, and hepatocytes activation markers; Gm5091 bound miR-27b, miR-23b, and miR-24, reducing their levels and alleviating AHF in mice.”

The correct text should read: “lncRNA Gm5091 was downregulated in hepatocytes of mouse during alcohol-associated hepatic ﬁbrosis (AHF), and alcohol-treated primary hepatocytes; lncRNA Gm5091 negatively regulated cell migration, reactive oxygen species levels, Il-1beta secretion, collagen I expression, and hepatocytes activation markers; Gm5091 bound miR-27b, miR-23b, and miR-24, reducing their levels and alleviating AHF in mice.”

